# Delta-6 desaturase (*Fads2*) deficiency alters triacylglycerol/fatty acid cycling in murine white adipose tissue

**DOI:** 10.1016/j.jlr.2023.100376

**Published:** 2023-04-19

**Authors:** Chenxuan Wang, Barbora Hucik, Ousseynou Sarr, Liam H. Brown, Kyle R.D. Wells, Keith R. Brunt, Manabu T. Nakamura, Ewa Harasim-Symbor, Adrian Chabowski, David M. Mutch

**Affiliations:** 1 Department of Human Health and Nutritional Sciences, University of Guelph, Guelph, ON, Canada; 2 Department of Pharmacology, Dalhousie University, Saint John, NB, Canada; 3 Department of Food Science and Human Nutrition, University of Illinois at Urbana-Champaign, Urbana, IL, USA; 4 Department of Physiology, Medical University of Bialystok, Bialystok, Poland

**Keywords:** delta-6 desaturase, *Fads2*, triacylglycerol, lipolysis, lipogenesis, omega-3 fatty acids

## Abstract

The Δ-6 desaturase (D6D) enzyme is not only critical for the synthesis of eicosapentaenoic acid (EPA) and docosahexaenoic acid (DHA) from α-linolenic acid (ALA), but recent evidence suggests that it also plays a role in adipocyte lipid metabolism and body weight; however, the mechanisms remain largely unexplored. The goal of this study was to investigate if a D6D deficiency would inhibit triacylglycerol storage and alter lipolytic and lipogenic pathways in mouse white adipose tissue (WAT) depots due to a disruption in EPA and DHA production. Male C57BL/6J D6D knockout (KO) and wild-type (WT) mice were fed either a 7% w/w lard or flax (ALA rich) diet for 21 weeks. Energy expenditure, physical activity, and substrate utilization were measured with metabolic caging. Inguinal and epididymal WAT depots were analyzed for changes in tissue weight, fatty acid composition, adipocyte size, and markers of lipogenesis, lipolysis, and insulin signaling. KO mice had lower body weight, higher serum nonesterified fatty acids, smaller WAT depots, and reduced adipocyte size compared to WT mice without altered food intake, energy expenditure, or physical activity, regardless of the diet. Markers of lipogenesis and lipolysis were more highly expressed in KO mice compared to WT mice in both depots, regardless of the diet. These changes were concomitant with lower basal insulin signaling in WAT. Collectively, a D6D deficiency alters triacylglycerol/fatty acid cycling in WAT by promoting lipolysis and reducing fatty acid re-esterification, which may be partially attributed to a reduction in WAT insulin signaling.

## Graphical abstract

Summary of key findings regarding the role of delta-6 desaturase on triacylglycerol/fatty acid cycling in white adipose tissue. Created with BioRender.com.

**Figure:**
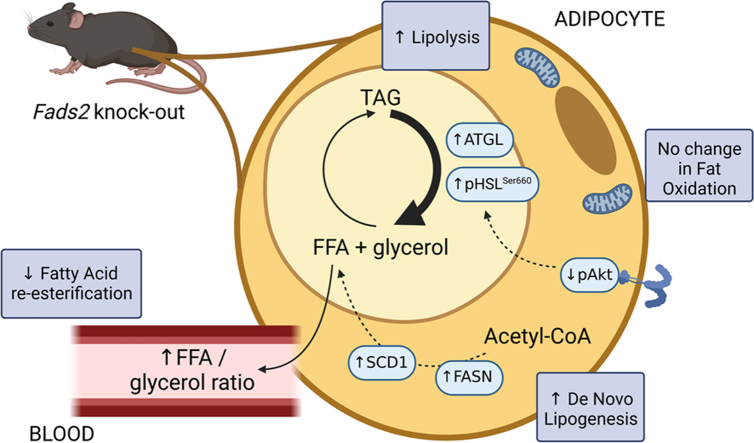

Omega-3 polyunsaturated fatty acids (N-3 PUFA) have been widely recognized for their roles in reducing disease risks and promoting health benefits (1, 2, 3). There are three major dietary n-3 PUFA, namely (ALA, 18:3n-3), eicosapentaenoic acid (EPA, 20:5n-3), and docosahexaenoic acid (DHA, 22:6n-3) (4). ALA is a plant-derived essential fatty acid that is obtained through the consumption of vegetable oils and nuts (4). EPA and DHA are marine-derived fatty acids that are primarily obtained through fish or fish oil consumption (4). However, a small amount of EPA and DHA can be endogenously synthesized from ALA through a pathway consisting of sequential desaturation and elongation steps (5, 6, 7). Briefly, Δ-6 desaturase (D6D; encoded by the *Fads2* gene) converts ALA to stearidonic acid (18:4n-3), followed by an elongation step to synthesize eicosatetraenoic acid (20:4n-3). Next, delta-5 desaturase (encoded by the *Fads1* gene) converts eicosatetraenoic acid into EPA, which can subsequently be used to generate DHA via further elongation and desaturation (5, 6, 7). Evidence shows that whole-body *Fads2* knockout (KO) mice have significantly lower levels of common ≥ C20 LC-PUFA in phospholipids (PLs) and triacylglycerols (TAGs) in various tissues compared to wild-type (WT) mice, indicating the key role of this enzyme in N3-PUFA synthesis (8, 9, 10).

White adipose tissue (WAT) is critical in whole-body energy homeostasis and is one of the major sites in which N-3 PUFA exert their effects (11, 12, 13). WAT stores excess energy in the form of TAG that can be broken down through hormonal regulation to release fatty acids in times of need (14). The excessive accumulation of lipids in WAT leads to obesity, which is generally associated with insulin resistance and a disruption in lipid metabolism pathways such as lipogenesis, lipolysis, and β-oxidation (15, 16, 17). Further, the various WAT depots in the body respond differently to metabolic stressors and thus have distinct contributions to disease endpoints (18). The two major distinct WAT depots are visceral adipose tissue and subcutaneous adipose tissue (19). As visceral adipose tissue mostly surrounds internal organs and is more metabolically active than subcutaneous adipose tissue, it therefore has a greater effect on the development of metabolic disorders when dysfunctional (20, 21).

The *Fads2* gene is expressed in mouse and human WAT, but little is known regarding its role in regulating WAT function. Previous research showed a lower body weight in *Fads2* KO mice when fed a diet deficient in EPA/DHA (9, 22, 23), and some of these studies reported smaller WAT mass and adipocyte size in KO mice (9, 23). However, the cellular mechanisms underlying these changes in WAT have not been extensively studied. Moreover, the adipose tissue phenotype observed in *Fads2* KO mice deficient in EPA/DHA would appear paradoxical to current knowledge regarding the benefits of these highly unsaturated omega-3 fatty acids. Indeed, animal and cell culture studies generally show a suppression effect of EPA and DHA on lipid accumulation in white adipocytes, which is associated with a reduction in lipogenesis and increases in TAG turnover and β-oxidation (11, 13, 24, 25, 26). Therefore, understanding the role of *Fads2* in the presence and absence of n-3 PUFA will provide new insights regarding how the D6D enzyme influences WAT lipid metabolism.

We recently investigated the effect of D6D inhibition on lipid accumulation in 3T3-L1 adipocytes during differentiation (27). We found that D6D-inhibited adipocytes had reduced TAG content and fatty acid re-esterification that was independent of changes in N-3 PUFA cellular content (27), suggesting a role for D6D in regulating adipocyte TAG/fatty acid cycling. Although the use of cultured cells is ideal to delineate molecular mechanisms under carefully controlled and uniform conditions, the translatability of these findings to an in vivo context is limited. Therefore, the present investigation examined the impact of a *Fads2* deficiency on pathways influencing TAG/fatty acid cycling in WAT depots collected from WT and *Fads2* KO mice fed an ALA-enriched diet.

## Materials and methods

### Animal housing and experimental diets

All experimental protocols were approved by the University of Guelph Animal Care Committee in accordance with the requirements of the Canadian Council on Animal Care. The *Fads2*^+/+^ WT and *Fads2*^−/−^ KO mice used in this study were generated by crossbreeding heterozygous (*Fads2*^+/−^) male and female C56BL/6J mice, which were obtained from the University of Illinois at Urbana-Champaign. Mice in breeding harems were fed a modified AIN-93G diet containing corn oil as the primary source of fat (CAT# D03090904P, Research Diets, Inc., New Brunswick, NJ). Offspring were genotyped at 17 days of age using Phire™ Tissue Direct PCR Master Mix (CAT#. F170S, Thermo Scientific) with the following primers: *Fads2* WT forward (5′-CGGTGGGAGGAGGAGTAGAAGAC-3′), *Fads2* WT reverse (3′-CCTCTCCCTGGTTACCTCCCTTC-5′), *Fads2* KO forward (5′-GCTATGACTGGGCACAACAG-3′) and *Fads2* KO reverse (3′-TTCGTCCAGATCATCCTGATC-5′). At 3 weeks of age, WT and KO male offspring were weaned and individually housed in conventional shoebox cages in a temperature- (22°C) and humidity-controlled room with a 12:12-h light-dark cycle. Mice were randomized to a lard control diet containing 7% (w/w) lard (CAT# D16090607, Research Diets, Inc.) or a flaxseed (ALA rich) diet containing 7% (w/w) flaxseed oil (CAT# D12041404, Research Diets, Inc.). Both diets were supplemented with 0.37% (w/w) ARASCO oil (DSM Nutritional Products Ltd., Canada Inc., Ayr, ON, Canada), which is rich in arachidonic acid (AA), to prevent AA deficiency–induced side effects such as dermatitis and intestinal ulcers in KO mice (8). Detailed diet composition is reported in supplemental Table S1, which was provided by the manufacturer. All mice had ad libitum access to their respective diets and water throughout the 21-week study. Body weight and food intake were measured weekly until 24 weeks of age.

### Intraperitoneal glucose and insulin tolerance tests

Intraperitoneal glucose tolerance test (IPGTT) and intraperitoneal insulin tolerance test (IPITT) were performed at 22 and 23 weeks of age, respectively. Mice were fasted for 6 h with ad libitum access to water prior to the tests. After measuring baseline blood glucose through the tip of the tail using a FreeStyle Lite glucometer (Abbott Diabetes Care Inc.), a glucose (2 g/kg body weight) or insulin (0.75 U/kg body weight) solution was administered via an intraperitoneal injection. Blood glucose levels were assessed at 15, 30, 45, 60, 90, and 120 min for IPGTT, or at 10, 20, 30, 45, 60, 90, and 120 min for IPITT. Area under the curve (AUC) or area above the curve (AAC) was determined as the total peak area above or below the baseline at *t* = 0 min using the trapezoid rule. The AAC for IPITT was also calculated for the 0–30 min time range to analyze glucose clearance without the confounding effect of hepatic glucose production.

### Metabolic caging

Three days after the IPITT, metabolic monitoring (nonexercise) was performed using a Comprehensive Lab Animal Monitoring System (CLAMS; Columbus Instruments, Columbus, Ohio). Mice were placed in the metabolic cages for 12 h to acclimatize. Subsequently, oxygen consumption (VO_2_, ml/min), carbon dioxide production (VCO_2_, ml/min), respiratory exchange ratio (RER, VCO_2_/VO_2_), energy expenditure (kcal/h; calculated as VO_2_ × (3.815 + (1.232 × RER)), and activity (infrared beam breaks in the *x* and *z* planes) were collected over a 24 h period that comprised a 12 h dark and a 12 h light cycle. The temperature and light-dark cycle remained the same during the CLAMS. Mice had free access to their respective diet and water throughout.

### Serum and tissue collection

At 24 weeks of age, mice were anesthetized with isoflurane followed by blood collection via cardiac puncture. Blood was allowed to clot for 30 min at room temperature and then centrifuged at 1,000 rcf for 15 min at 4°C. Serum was collected and flash frozen in liquid nitrogen. The death of mice was ensured by decapitation. Inguinal white adipose tissue (iWAT) and epididymal white adipose tissue (eWAT) were excised and weighed. A small piece from each WAT sample was separated and fixed in 4% paraformaldehyde solution at 4°C for 24 h, and then washed with 1× PBS and stored in 70% ethanol at 4°C for histological processing. The rest of the tissue was flash frozen in liquid nitrogen immediately after collection. Serum and tissue samples were stored at −80°C until further analysis.

### Serum nonesterified fatty acid, glycerol, and insulin measurements

Serum nonesterified fatty acid (NEFA) content was measured with a NEFA HR Series NEFA-HR kit (CAT#: 999-34691, 995-34791, 991-34891 & 993-35191, FUJIFILM Medical Systems, VA), glycerol content was measured with a Free Glycerol Reagent kit (CAT#: F6428, Sigma-Aldrich), and insulin was measured using a Mouse Insulin ELISA kit (CAT#: 10124701, Mercodia AB), all according to manufacturer's instructions.

### Histological processing, hematoxylin and eosin staining, and adipocyte size measurement

Fixed adipose tissues were placed in a tissue processor (CAT#: A78400111, Shandon Excelsior ES®, Thermo Scientific), which performed automatic 45-min submersions in increasing isopropanol concentrations at 70%, 85%, 90%, 95%, and 100% under vacuum conditions, followed by three 45-min submersion steps in xylene and three 45-min submersion steps in paraffin wax. Tissues were embedded in paraffin wax on an embedding workstation (CAT#: A81000101, HistoStar™, Thermo Scientific). Embedded tissue blocks were sectioned at 5.0 μm using a rotary microtome (CAT#: RM2255, Leica Biosystems). Sections were allowed to smoothen in a water bath before transferring to microscope slides. Slides were dried and fixed overnight on a 37°C hot plate.

Hematoxylin and eosin staining was performed by submerging the fixed slides in xylene three times with 2 min each, followed by three 2-min submersion steps in 100% isopropanol, a 2-min submersion step in 70% isopropanol, and a 2-min submersion step in deionized water. Next, slides were transferred to Harris Modified Hematoxylin stain with acetic acid for 10 min, followed by a thorough rinsing in deionized water. The slides were then dipped eight times in an acid alcohol solution (70% isopropanol & 1% hydrochloric acid). After a thorough wash in deionized water, the slides were dipped in ammonia water until the tissue turned blue, followed by another washing step in deionized water. Next, the slides were dipped six times in 70% isopropanol before being placed in an eosin staining solution (0.2% eosin Y solution, 60% ethanol, and 0.5% glacial acetic acid) for 30 s, followed by a dehydration step via three 2-min 100% isopropanol submersions and a clear step via three 2-min xylene submersions. Finally, slides were coverslipped using Cytoseal™ XYL mounting medium (CAT#: 8312-16E, Thermo Scientific) and dried overnight in a fume hood.

Slides were scanned using a Zeiss Microscope at 5× magnification. Adipocyte sizes were measured by Adiposoft software. Data were analyzed following a protocol adapted from Parlee *et al.* (28). One hundred adipocytes were randomly selected from each sample to calculate average adipocyte sizes by an individual blinded to the mouse IDs.

### Lipid fraction analyses

Lipid extraction and fractionation protocols were adapted from previously reported studies (29, 30). In brief, WAT samples were homogenized, and lipids were extracted in a chloroform-methanol solution (2:1 v/v) containing butylated hydroxytoluene as an antioxidant and heptadecanoic acid as an internal standard. After overnight incubation, water was added followed by centrifugation at 3,000 rpm for 10 min. The lower layer was collected and separated using thin-layer chromatography on silica gel plates (Silica Plate 60, 0.25 mm; Merck, Darmstadt, Germany) with a heptane/isopropyl ether/acetic acid (60:40:3, v/v/v) resolving solution. PL, TAG, and diacylglycerol (DAG) fractions were visualized under a UV light. Each fraction band was then scraped and collected. The DAG fraction was eluted using a chloroform/methanol solution (9:1, v/v), and the organic phase was collected and added with 14% boron trifluoride-methanol (BF_3_) solution for transmethylation using the Christie method (31). TAG and PL fractions were eluted with diethyl ether/hexane (1:1, v/v) and chloroform/methanol/water (5:5:1, v/v/v) solutions, respectively, prior to transmethylation. Fatty acid methyl esters were extracted using a pentane, which was then evaporated under a stream of nitrogen gas. Subsequently, samples were dissolved in hexane and analyzed with a Hewlett-Packard 5,890 Series II gas chromatograph, an Agilent J&W CP-Sil 88 capillary column (50 m × 0.25 mm inner diameter), and a flame-ionization detector (Agilent Technologies, Santa Clara, CA). Fatty acid profiles are reported as either the total μmol fatty acid (i.e., the sum of each fatty acid across TAG, PL, and DAG fractions) per g of tissue or as a % of total fatty acids in each lipid fraction.

### RNA and protein extraction

Total RNA from iWAT and eWAT samples was extracted using the RNeasy Mini Kit (CAT#. 74106, Qiagen, Mississauga, ON, CA) with an on-column DNase digestion step, as per manufacturer's instructions. RNA concentration and purity were determined using a NanoDrop™ 2,000 Spectrophotometer (Thermo Fisher Scientific, Wilmington). Only samples with ratios (A260/A230 and A260/A280) between 1.8 and 2.2 were used for cDNA synthesis.

Protein was recovered from the flow-through eluate retained during the RNA extraction process, as described in a previous method for dual RNA and protein extraction using the Qiagen RNeasy Mini Kit (32). In brief, 4× volume of ice-cold acetone was added into each sample eluate followed by a short vortex. The mixture was left overnight at −20°C to allow for protein precipitation, which was then centrifuged at 15,000 rcf for 10 min. After removal of the supernatant, the protein pellet was air-dried and then resuspended in a lysis buffer. Protein concentration was determined using a NanoDrop™ 2,000 Spectrophotometer with a measurement of A280.

### Reverse transcription quantitative PCR

One μg of RNA from each sample was reverse transcribed to cDNA using the Applied Biosystems™ High-Capacity cDNA Reverse Transcription Kit (CAT#: 4368814, Thermo Fisher Scientific), as per manufacturer's instructions. Primer design and reverse transcription quantitative PCR were carried out as described previously (27). *Nono* expression was unchanged between groups and was used as a housekeeping gene. Primer sequences are listed in supplemental Table S2. Results were quantified using the ΔΔCt method.

### Western blotting

Western blotting was carried out as described previously (27). Both αTubulin and Ponceau were used as loading controls. All quantitative data were calculated using Ponceau control. αTubulin is presented as a control in representative images. The following primary antibodies used in this study were obtained from Cell Signaling Technology: PPAR-γ (CAT#: 2443), SCD1 (CAT#: 2438), FASN (CAT#: 3180S), ACC (CAT#: 3662), phospho-ACC Ser-79 (Cat#: 3661), ATGL (CAT#: 2439), phospho-HSL Ser-660 (CAT#: 4126), phospho-HSL Ser-563 (CAT#: 4139), phospho-HSL Ser-565 (CAT#: 4137), HSL (CAT#: 4107), AKT (CAT#: 9272), phospho-AKT Ser-473 (CAT#: 9271), and phospho-AKT Thr-308 (CAT#: 9275). The primary antibodies obtained from Abcam were DGAT2 (CAT#: 237613) and αTubulin (CAT#: ab7291). Other primary antibodies include SREBP1 (CAT#: 2215, Novus Biologicals) and PEPCK1 (CAT#: 10004943, Cayman Chemical). The secondary antibodies used were Goat Anti-Rabbit IgG (H + L)-HRP conjugate (CAT#: 1706515, Bio-Rad Laboratories), and Goat Anti-Mouse IgG (H + L)-HRP conjugate (CAT#: 1706516, Bio-Rad Laboratories).

### Statistical analyses

All analyses were run using GraphPad Prism 8.0.1 (GraphPad Software Incorporated, La Jolla). Data were analyzed by one-way ANOVA or two-way ANOVA for main effects of diet (P_diet_) and genotype (P_genotype_), as well as the diet × genotype interaction (P_Interaction_), followed by a posthoc Tukey's multiple comparison test. For metabolic caging data, VO_2_, VCO_2_, and energy expenditure were also analyzed with an ANCOVA to account for body weight as a variable factor. A *P* ≤ 0.05 was used as the threshold for statistical significance. Results are presented as mean ± standard deviation (SD).

## Results

### *Fads2* genotype affects body weight without altering food intake

**Fig. 1:**
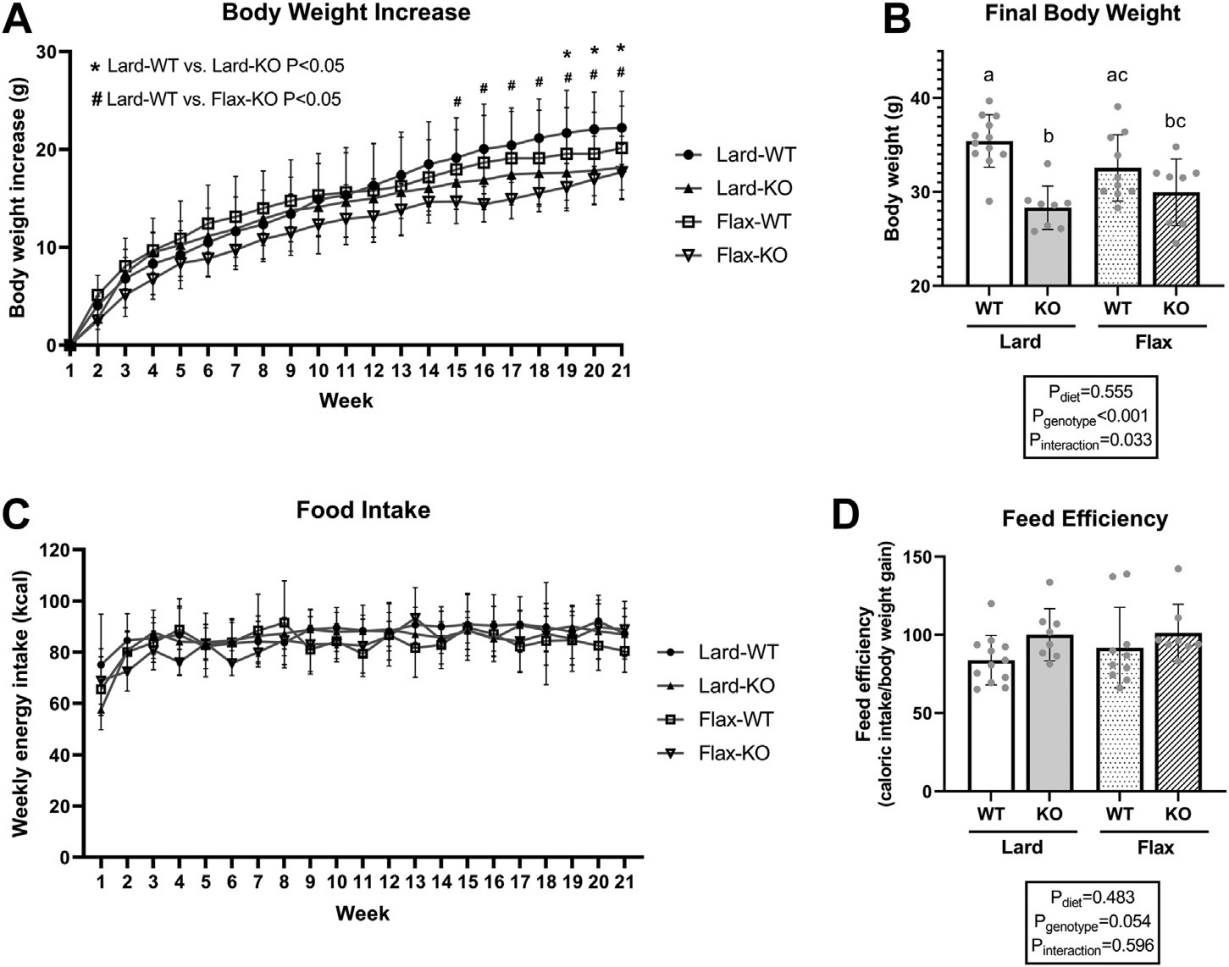
Body weight, food intake, and feed efficiency in WT and *Fads2* KO mice fed a lard or flax diet. A: Body weight gain during the 21-week feeding study. B: Final body weight measured at the end of the 21-week feeding study. C: Weekly food intake (kcal). D: Feed efficiency calculated as total caloric intake/total body weight gain during the 21-week feeding study. All data are presented as mean ± SD (n = 8–12 mice/group). Body weight, food intake, and feed efficiency were analyzed by 2-way ANOVA for main effects (P_diet_ & P_genotype_) and interaction (P_Interaction_) followed by a Tukey's posthoc test. Different letters over bars indicate a significant difference (*P* < 0.05) between groups.

We first assessed if the *Fads2* genotype or experimental diets affected body weight and energy intake. In general, the *Fads2* genotype had a significant main effect on final body weight (Fig. 1B, *P*_genotype_ < 0.001), while diet had no effect (Fig. 1B, *P*_diet_ = 0.555). Specifically, when fed with a lard diet, KO mice gained less body weight compared to WT mice from week 15 to termination (Fig. 1A), which aligned with a lower final body weight (Fig. 1B). No statistical differences in weight were observed between Flax-WT and Flax-KO groups, despite the visual trend seen in the data suggesting that Flax-KO mice have lower body weights compared to Flax-WT mice (Fig. 1A, B). No differences were observed in weekly food intake (Fig. 1C) between the four groups; however, a strong trend was observed suggesting a difference in feed efficiency between WT and KO mice (Fig. 1D, *P*_genotype_ = 0.054).

### Neither *Fads2* genotype nor diet affect glucose homeostasis, whole body substrate utilization, or physical activity

**Fig. 2:**
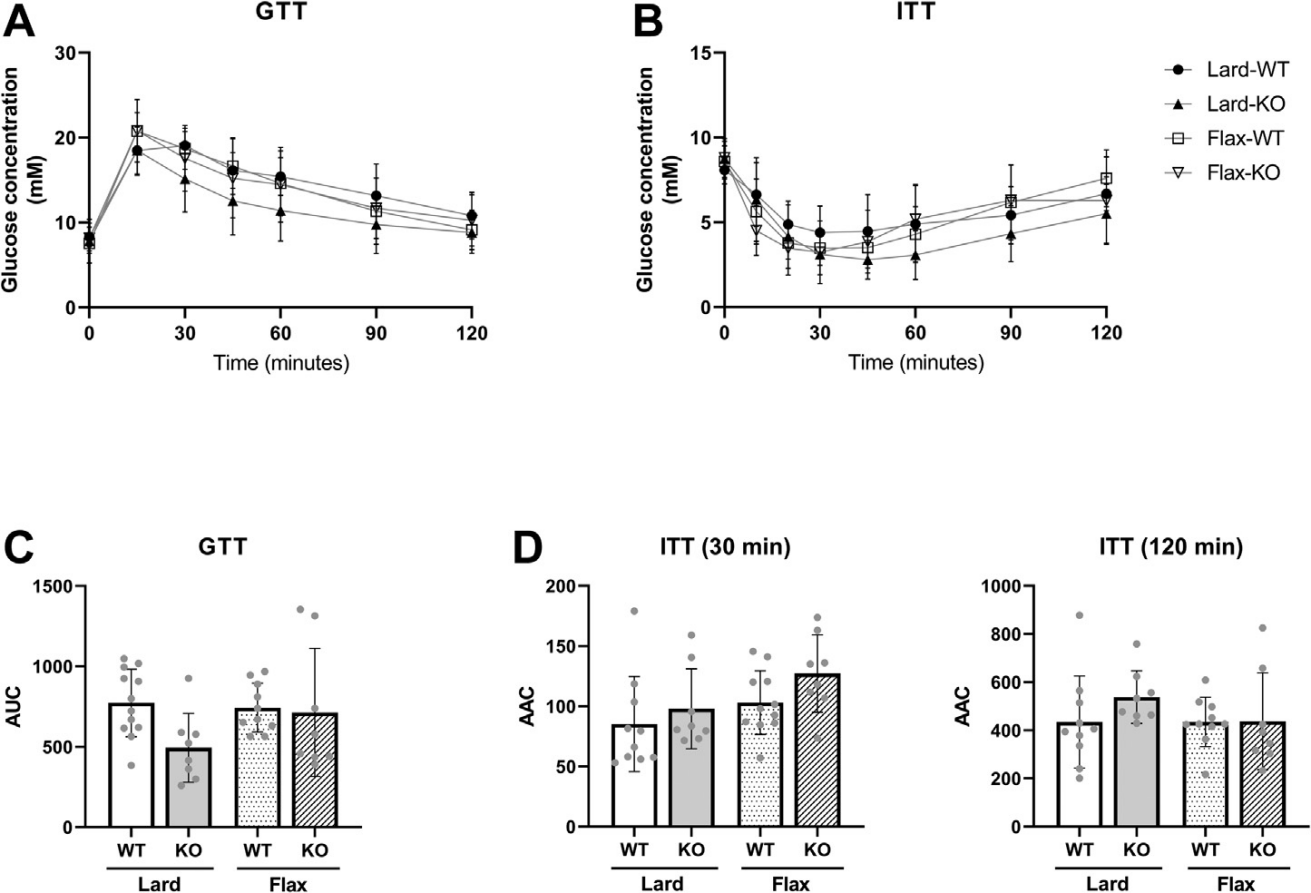
Glucose and insulin tolerance in WT and *Fads2* KO mice fed a lard or flax diet. Blood glucose was measured after a 6 h fast, before intraperitoneal injection of (A) glucose (2 g/kg body weight) or (B) insulin (0.75 U/kg body weight), and during the following two-hour period postinjection. The AUC is shown for glucose tolerance (C), while the AAC is shown for insulin during the first 30 min and the entire two-hour period (D). AUC and AAC were determined as the total peak area above or below the baseline at t = 0 min using the trapezoid rule, respectively. All data are presented as mean ± SD (n = 8–12 mice/group). AUC and AAC were analyzed by 2-way ANOVA followed by a Tukey's posthoc test. No significant differences (*P* < 0.05) were found.

**Fig. 3:**
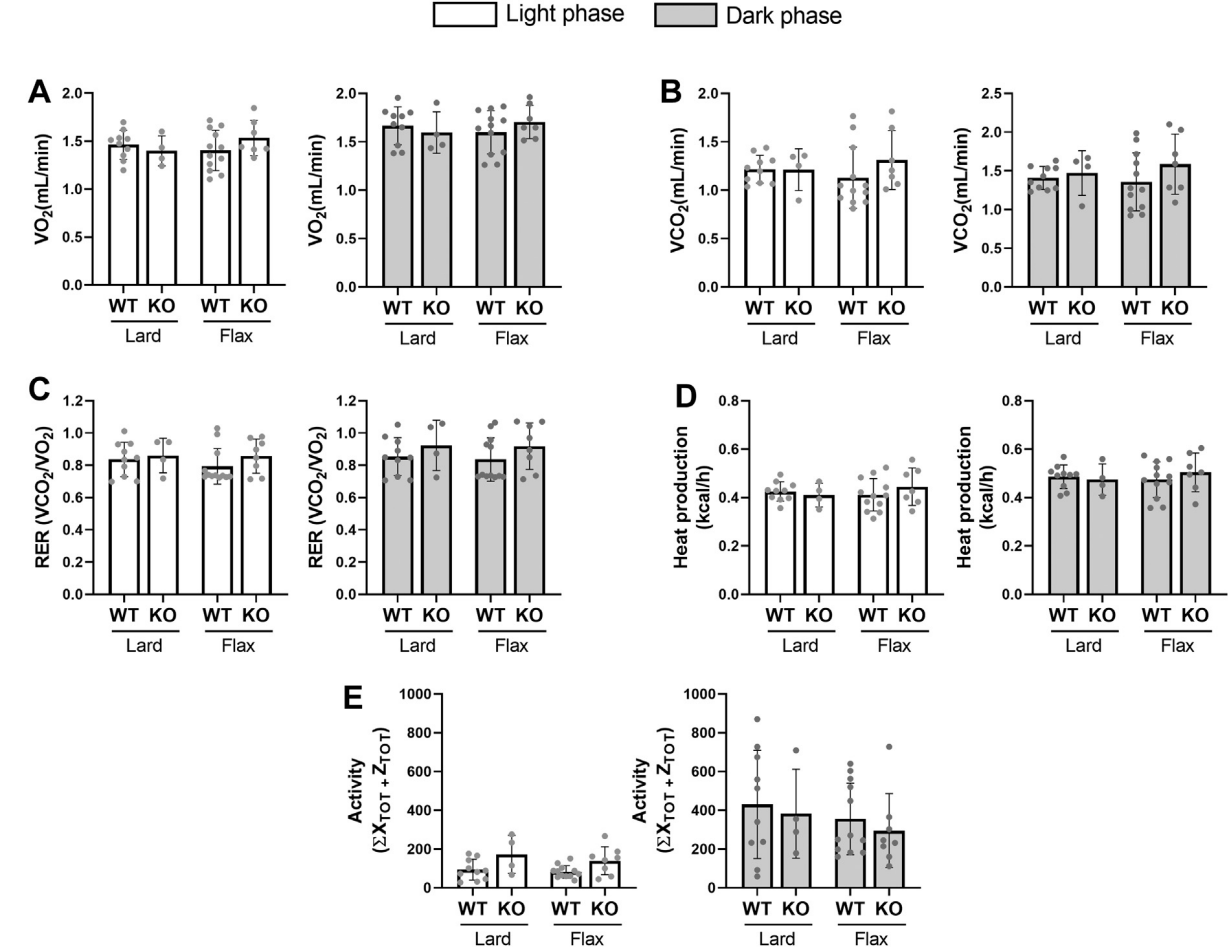
Resting energy expenditure and physical activity of WT and *Fads2* KO mice fed a lard or flax diet. Mice were placed in a CLAMS for 12 h to acclimatize. (A) VO_2_, (B) VCO_2_, (C) RER, (D) energy expenditure, and (E) activity (infrared beams breaks in the *x* and *z* planes) were collected in 12 h light and 12 h dark phases. All data are presented as mean ± SD (n = 8–12 mice/group). Data were analyzed by 2-way ANOVA followed by a Tukey's posthoc test. No main effects or significant differences between groups (*P* < 0.05) were found.

We next investigated the effects of the *Fads2* genotype and diets on various whole-body measurements. No significant differences were found in glucose tolerance and insulin response between the four groups, as shown by IPGTT and IPITT, respectively (Fig. 2A–D). Similarly, no significant differences were observed in VO_2_, VCO_2_, RER, or energy expenditure in either the light or dark phases (Fig. 3). As expected, physical activity was higher in all mice during the dark phase. An ANCOVA was subsequently performed to account for body weight as a variable in VO_2_, VCO_2_, and energy expenditure; however, no differences were detected (data not shown).

### Lower WAT depot weight and adipocyte size in *Fads2* KO mice

**Fig. 4:**
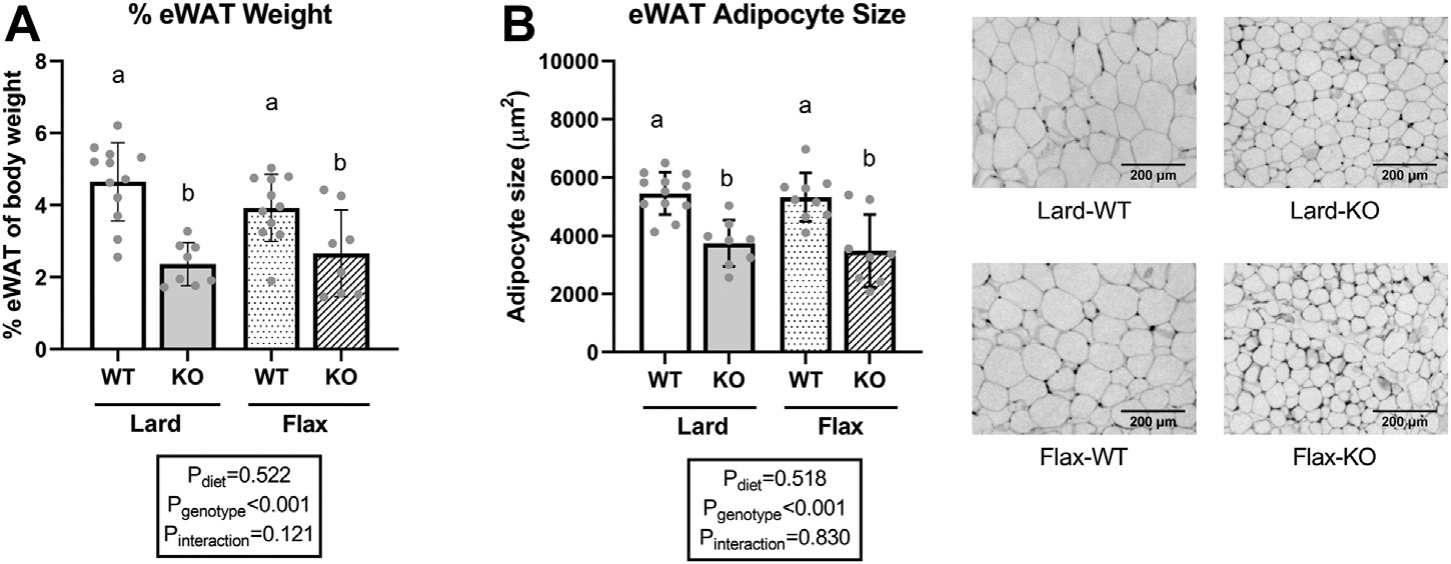
Tissue weights and adipocyte sizes of eWAT in WT and *Fads2* KO mice fed a lard or flax diet. A: eWAT tissue weight was calculated as % of body weight. B: eWAT adipocyte size and representative images. All data are presented as mean ± SD (n = 8–12 mice/group). Data were analyzed by 2-way ANOVA for main effects (P_diet_ & P_genotype_) and interaction (P_Interaction_) followed by a Tukey's posthoc test. *Different letters* indicate a significant difference (*P* < 0.05) between groups.

Since we observed a highly significant main effect for genotype on body weights (Fig. 1B), we next examined tissue weight and adipocyte size for visceral eWAT and subcutaneous iWAT in *Fads2* WT and KO mice. In general, *Fads2* genotype had a significant main effect on both tissue weight and adipocyte size in both depots (Fig. 4; supplemental Fig. S1), whereas diet generally showed no main effect except for % iWAT weight (supplemental Fig. S1A, *P*_diet_ = 0.036). Specifically, KO mice had lower eWAT and iWAT weights compared to WT mice in both diet groups. Average adipocyte size in eWAT aligned with the lower tissue weight. In iWAT, Lard-KO mice had smaller adipocytes compared to Lard-WT mice; however, this difference was not observed in the flax groups (supplemental Fig. S1B).

### Higher serum NEFA and NEFA/glycerol ratio in *Fads2* KO mice

**Fig. 5:**
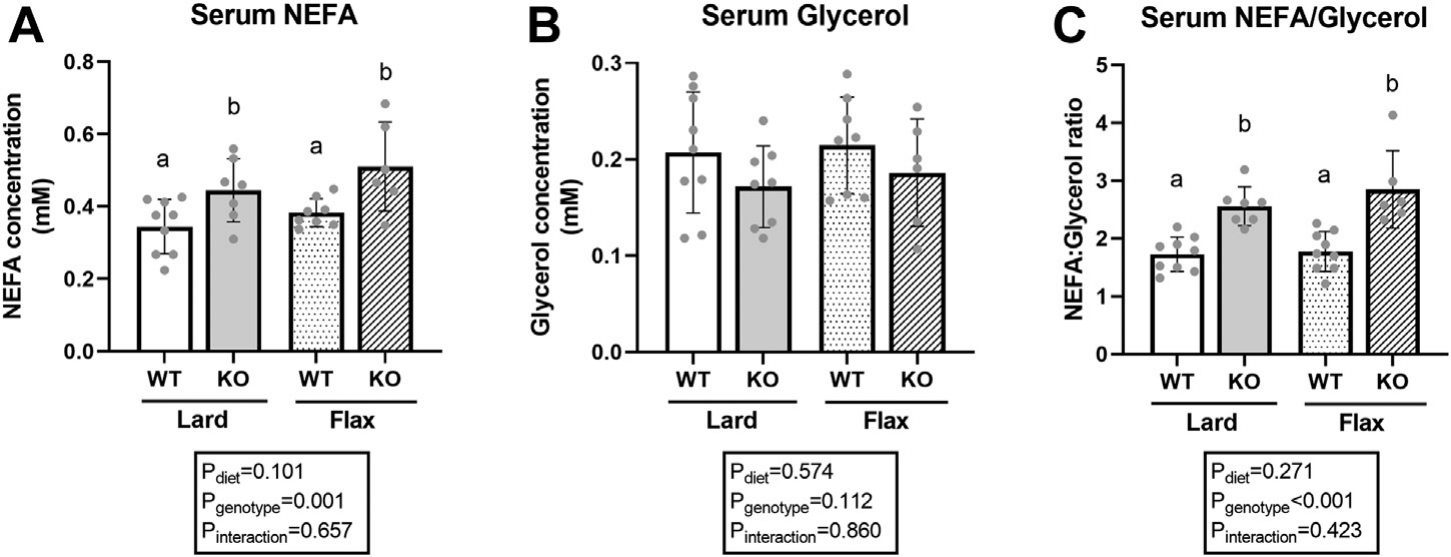
Serum NEFA (A), glycerol (B), and NEFA/glycerol ratio (C) in WT and *Fads2* KO mice fed a lard or flax diet. All data are presented as mean ± SD (n = 6–8 mice/group). Data were analyzed by 2-way ANOVA for main effects (P_diet_ & P_genotype_) and interaction (P_Interaction_) followed by a Tukey's posthoc test. *Different letters* indicate a significant difference (*P* < 0.05) between groups.

Given the lower WAT weights observed in *Fads2* KO mice, we analyzed circulating NEFA, glycerol, and the NEFA/glycerol ratio in mice. The *Fads2* genotype showed a significant main effect on serum NEFA (Fig. 5A, *P*_genotype_ = 0.001) and the NEFA/glycerol ratio (Fig. 5C, *P*_genotype_ < 0.001), but not on serum glycerol (Fig. 5B, *P*_genotype_ = 0.112). Specifically, KO mice had a higher serum NEFA and NEFA/glycerol ratio compared to WT mice in both diet groups (Fig. 5A, C). No significant main effect of diet was observed with any of these markers.

### *Fads2* deficiency alters TAG N-3 PUFA composition in both eWAT and iWAT

**Fig. 6:**
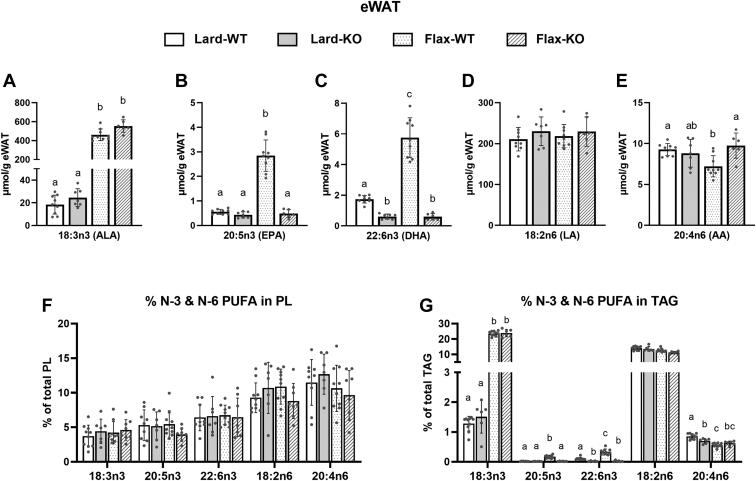
N-3 and N-6 fatty acid composition in eWAT from WT and *Fads2* KO mice fed a lard or flax diet. The complete fatty acid composition of PL, TAG, and DAG fractions is reported in supplemental Table S3. Total μmol per g tissue of (A) ALA, (B) EPA, (C) DHA, (D) LA, and (E) AA were calculated by summing the values in PL, TAG, and DAG fractions for each fatty acid. N-3 and N-6 PUFAs in PL (F) and TAG (G) are presented as % of total fatty acids in each respective fraction. All data are reported as mean ± SD (n = 6–8 mice/group). Data were analyzed by one-way ANOVA with Tukey’s posthoc test. *Different letters* indicate a significant difference (*P* < 0.05) between groups.

We next investigated the impact of a *Fads2* deficiency on fatty acid composition in PL, TAG, and DAG fractions of eWAT and iWAT (supplemental Tables S3 and S4). Major N-3 and N-6 PUFA content are shown for eWAT (Fig. 6) and iWAT (supplemental Fig. S2), reported as total μmol/g tissue, as well as % in TAG and PL fractions. As expected, mice fed a flax diet had significantly higher total ALA content regardless of the genotype in both eWAT and iWAT. When fed a flax diet, KO mice had significantly lower total EPA and DHA content compared to WT mice in both WAT depots. Both WT and KO mice fed a lard diet showed a similar total EPA content to Flax-KO mice. However, Lard-KO mice and Flax-KO mice had similarly low levels of DHA, which was significantly lower than Lard-WT mice. In both WAT depots, the differences in total ALA, EPA, and DHA levels were reflected in the TAG fraction but not the PL fraction. No differences were observed in total LA and AA between the four groups, except for lower total AA in the eWAT of Flax-WT mice compared to the other three groups (Fig. 6E).

### Lipogenic markers are higher in eWAT and iWAT of *Fads2* KO mice

**Fig. 7:**
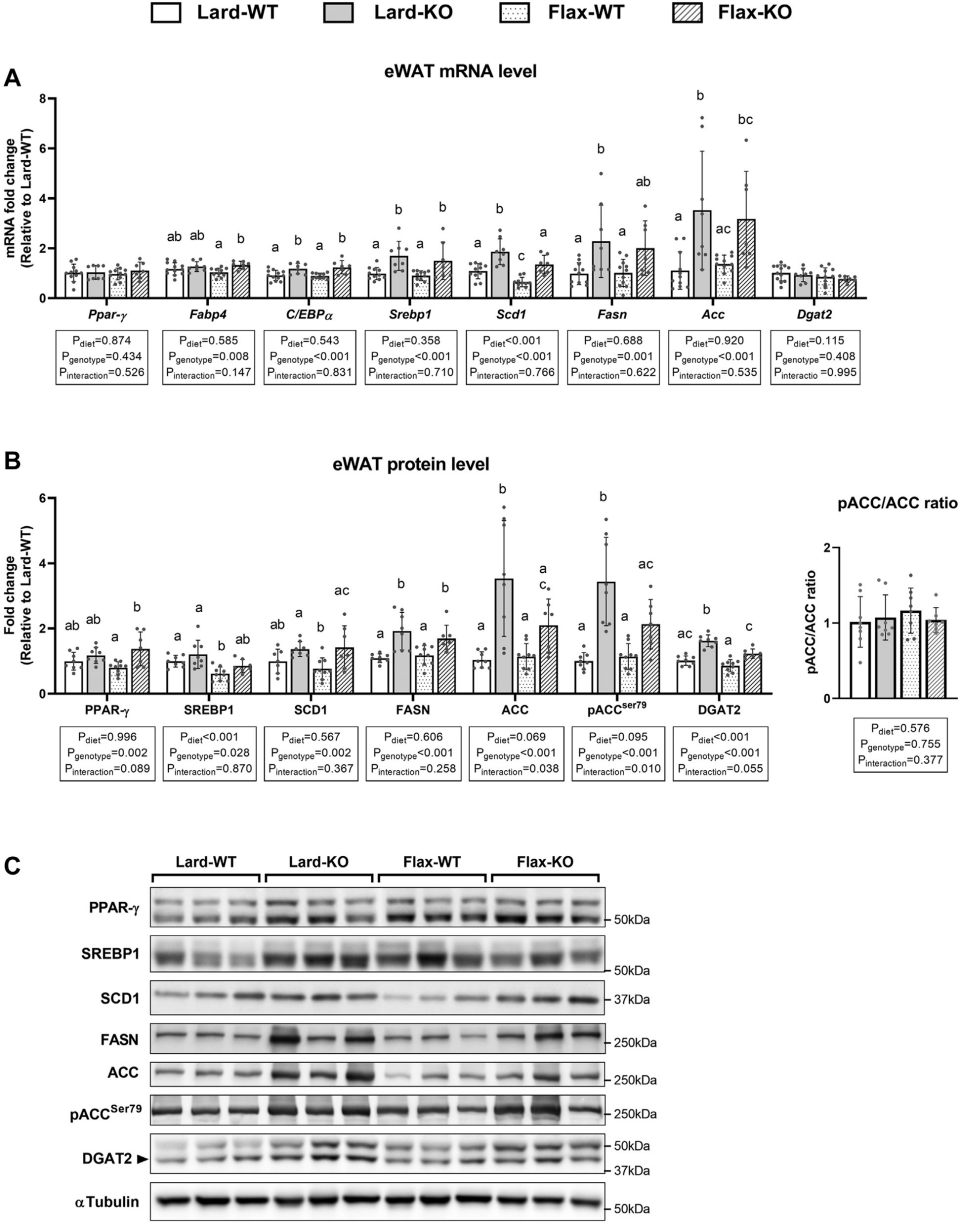
Lipogenic markers in eWAT from WT and *Fads2* KO mice fed a lard or flax diet. A: RT-qPCR analysis of markers involved in adipogenesis and lipogenesis. B: Western blot analyses of markers involved in lipogenesis. Data are presented as fold changes compared to the Lard-WT group. C: Representative images from Western blotting shown for three mice/group. αTubulin is shown as a representative loading control. All data are presented as mean ± SD (n = 7–12 mice/group). Data were analyzed by 2-way ANOVA for main effects (P_diet_ & P_genotype_) and interaction (P_Interaction_) followed by a Tukey's posthoc test. *Different letters* indicate a significant difference (*P* < 0.05) between groups.

Due to the lower WAT weights observed in *Fads2* KO mice, we subsequently examined common markers of adipogenesis and lipogenesis in eWAT (Fig. 7) and iWAT (supplemental Fig. S3). We first examined mRNA levels. In general, no main effects of diet were observed for any of the genes examined. However, the *Fads2* genotype showed a significant main effect on most lipogenic markers in both WAT depots apart for *Dgat2*. Specifically, *Srebp1*, *Fasn*, *Acc*, and *Scd1* expression was upregulated in KO mice compared to WT mice in both diet groups, except for *Srebp1* in iWAT of mice fed a flax diet (supplemental Fig. S3A). Further, KO mice showed either unchanged or higher mRNA levels of *Ppar-γ*, *Fabp4*, and *C/EBPα* compared to WT mice in both diet groups, suggesting that the smaller adipocyte phenotype observed with a *Fads2* deficiency is not due to a general impairment in adipogenesis.

Changes at the mRNA level are not always reflected at the protein level; therefore, we also examined the protein abundance of lipogenic makers in the two WAT depots. Similar to what we observed at the mRNA level, there was a minimal effect of diet on most of the lipogenic markers in both depots, except for SREBP1 and DGAT2 in eWAT. In contrast, there was a significant main effect of the *Fads2* genotype on most of the lipogenic markers, except for DGAT2 in iWAT (Fig. 7B, C; supplemental Fig. S3B, C). Specifically, FASN and DGAT2 were higher in KO mouse eWAT compared to WT mice in both diet groups, whereas SCD1 in KO mice was higher only in the flax group, and ACC and pACC^Ser79^ in KO mice were higher only in lard group (Fig. 7B). Both ACC and pACC^Ser79^ in eWAT appeared higher in Flax-KO mice compared to Flax-WT mice, although these differences were not statistically significant in the posthoc analysis (*P* = 0.25 and *P* = 0.21, respectively; Fig. 7B). In iWAT, SREBP1, FASN, ACC, and pACC^Ser79^ were all higher in KO mice compared to WT mice in both diet groups, whereas SCD1 in KO mice was higher only in the flax group (supplemental Fig. S3). In addition, no significant differences were observed in the pACC^Ser79^/ACC ratio between groups in either WAT depot. In alignment with the *Ppar-γ* mRNA levels, no difference in PPAR-γ protein was observed apart for a slight increase in the eWAT of Flax-KO mice (Fig. 7B).

### Lipolytic markers are higher in eWAT and iWAT of *Fads2* KO mice

**Fig. 8:**
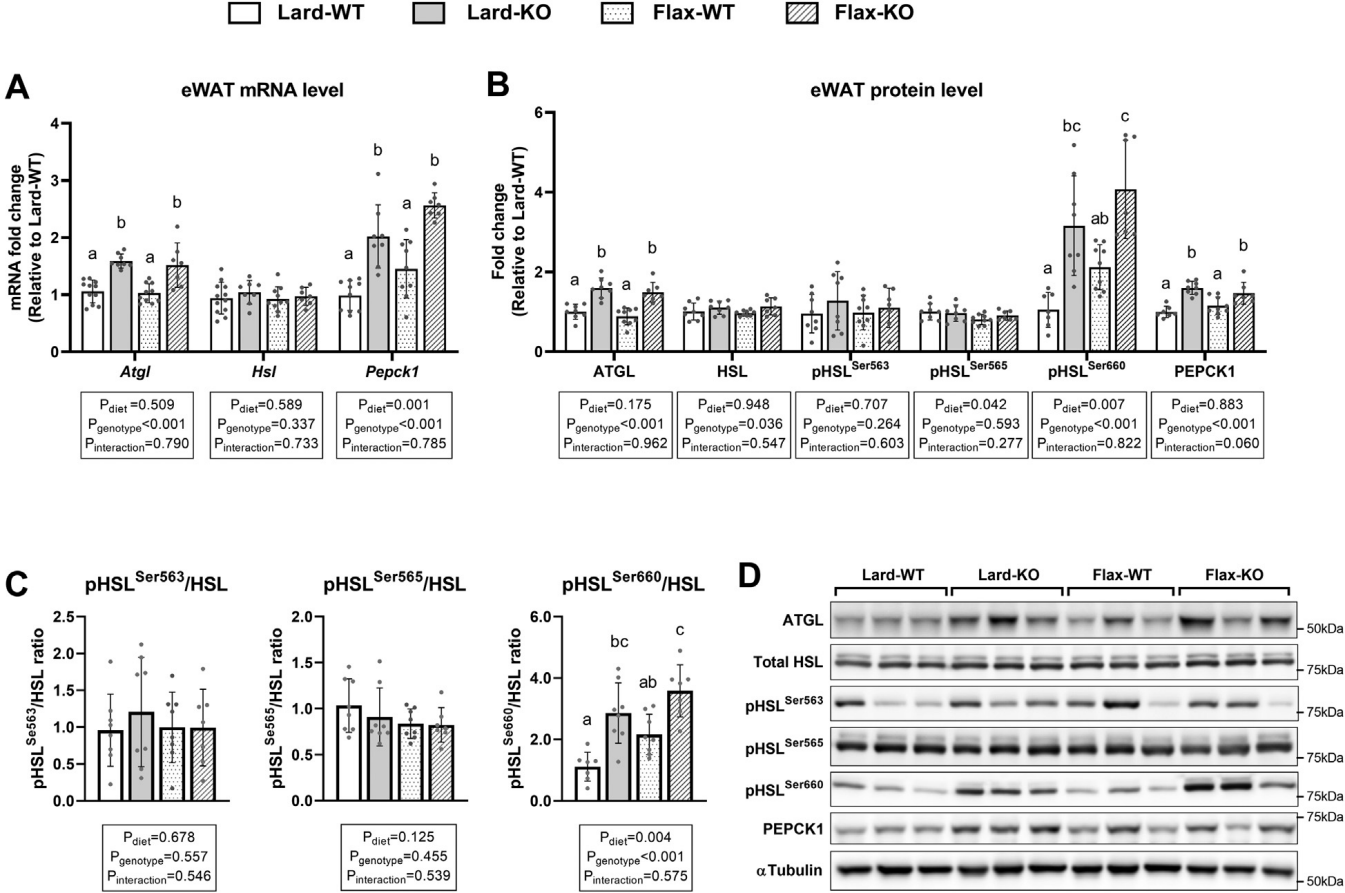
Lipolytic markers in eWAT from WT and *Fads2* KO mice fed a lard or flax diet. A: RT-qPCR analysis of markers involved in lipolysis. B: Western blot analyses of markers involved in lipolysis. Data are presented as fold changes against Lard-WT group. C: Ratios of pHSL to total HSL. D: Representative images from Western blotting shown for three mice/group. αTubulin is shown as a representative loading control and is the same as the αTubulin presented in Fig. 7C. All data are presented as mean ± SD (n = 7–12 mice/group). Data were analyzed by 2-way ANOVA for main effects (P_diet_ & P_genotype_) and interaction (P_Interaction_) followed by a Tukey's posthoc test. *Different letters* above bars indicate a significant difference (*P* < 0.05) between groups.

Since we observed higher circulating NEFA in *Fads2 KO* mice, as well as lower WAT tissue weights, we next examined common markers of lipolysis. We also tested PEPCK1, which is a marker for fatty acid re-esterification that is important for TAG/fatty acid cycling in WAT. In eWAT from KO mice, ATGL and PEPCK1 were upregulated at both the mRNA and protein level compared to WT mice regardless of the diet (Fig. 8A, B). In iWAT from KO mice, only ATGL was consistently upregulated at the protein level compared to WT in both diet groups (supplemental Fig. S4). Although *Atgl* mRNA appeared higher in the iWAT of KO mice, it did not reach statistical significance when compared to WT mice; however, the *Fads2* genotype showed a significant main effect (*P*_genotype_ = 0.01, supplemental Fig. S4A). Similar to eWAT, *Pepck1* in iWAT of KO mice was higher compared to WT mice at the mRNA level regardless of diet, but a significant increase at the protein level was only observed in the lard group following posthoc analysis. In both eWAT and iWAT, no differences were observed in *Hsl* mRNA and total HSL protein levels across all groups (Fig. 8A, B; supplemental Fig. S4A, B). However, the activation site pHSL^Ser660^ and the pHSL^Ser660^/HSL ratio were both significantly higher in KO mice compared to WT mice regardless of the diet (Fig. 8B, C; supplemental Fig. S4B, C). No differences were found for pHSL^Ser563^, pHSL ^Ser563^/HSL ratio, pHSL^Ser565^, and pHSL^Ser565^/HSL ratio in either WAT depot.

### Increased markers of lipolysis are associated with lower insulin signaling in WAT

**Fig. 9:**
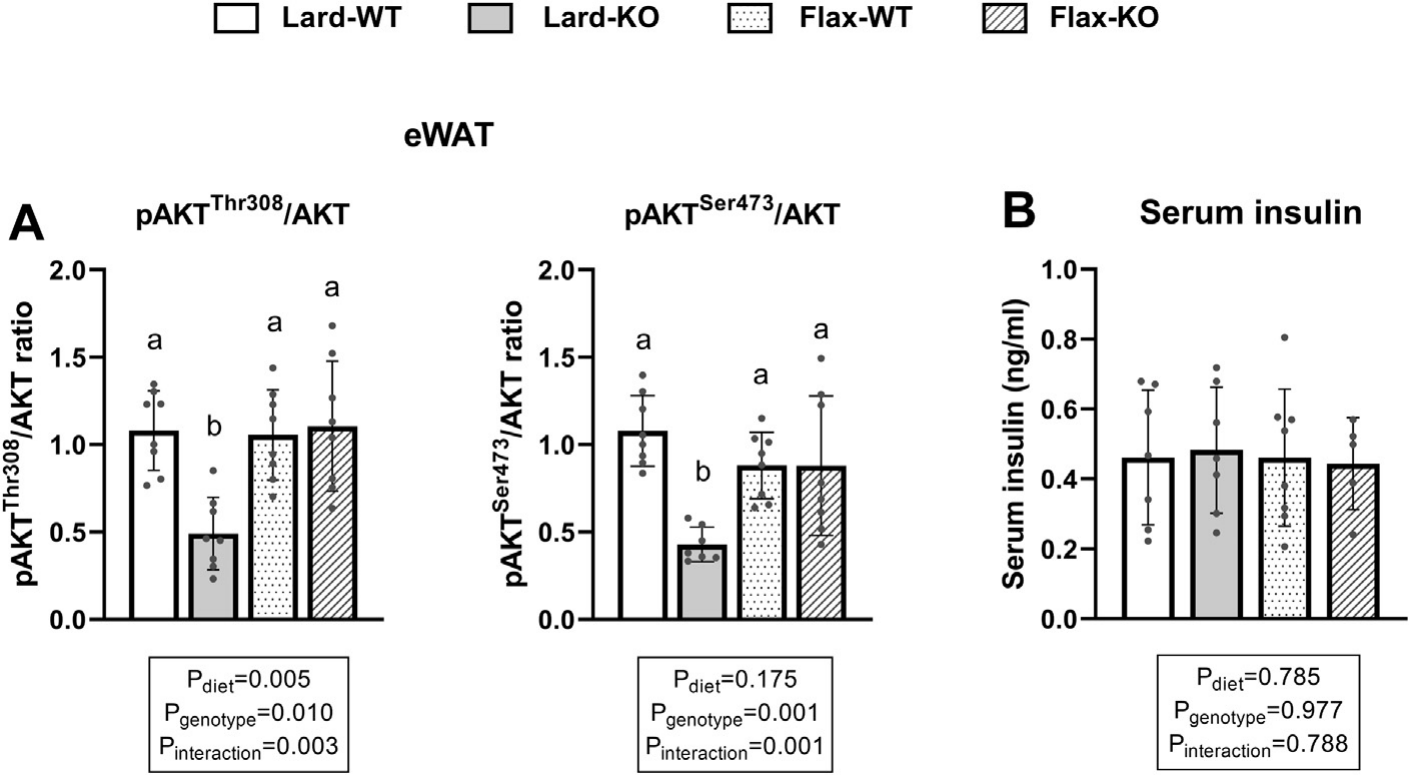
Insulin signaling markers in eWAT and serum insulin levels in WT and *Fads2* KO mice fed a lard or flax diet. A: Western blot analyses of two pAKT/AKT ratios in eWAT. B: Serum insulin concentrations. All data are presented as mean ± SD (n = 7–9 mice/group). Data were analyzed by 2-way ANOVA for main effects (P_diet_ & P_genotype_) and interaction (P_Interaction_) followed by a Tukey's posthoc test. *Different letters* above bars indicate a significant difference (*P* < 0.05) between groups.

Following our observation that ATGL was upregulated and HSL activated in *Fads2* KO mice, we investigated if these increases were associated with lower insulin signaling in WAT depots in the basal state. In mice fed a lard diet, KO mice had lower pAKT^Thr308^/AKT and pAKT^Ser473^/AKT ratios compared to WT mice in both iWAT and eWAT (Fig. 9; supplemental Fig. S5). However, when mice were fed with a flax diet, no differences were observed between WT and KO mice except for a lower pAKT^Thr308^/AKT ratio in iWAT (supplemental Fig. S5A). Notably, the genotype × diet interactions were significant for both pAKT/AKT ratios in both depots. To examine if the reduced insulin signaling markers were related to a reduced circulating insulin, we also measured serum insulin levels. No differences were observed in serum insulin between the various groups (Fig. 9). No significant correlations were found between either of the two pAKT/AKT ratios and serum insulin levels (data not shown).

## Discussion

Using whole-body *Fads2* KO mice, the present study provides new insight regarding a role for *Fads2* in the regulation of TAG/fatty acid cycling in WAT. Overall, we found that a *Fads2* deficiency lowered TAG content and fatty acid re-esterification while promoting lipolysis in WAT depots, which may be partially attributed to impaired WAT insulin signaling. Moreover, we found that diet had little-to-no impact on the changes observed in *Fads2* KO mice. The specific key findings from this study include: *1*) KO mice had lower body weight, smaller WAT depots, and smaller adipocytes compared to WT mice without showing alterations in food intake, energy expenditure, or physical activity, regardless of the diet, *2*) *Fads2* deficiency-induced changes in total ALA, EPA, and DHA levels were reflected in the TAG fraction but not in the PL fraction in both depots, *3*) *Fads2* KO mice had increased markers of lipogenesis and lipolysis in WAT depots, as well as higher levels of serum NEFA and NEFA/glycerol ratio, compared to WT mice, and *4*) *Fads2* KO mice fed a lard diet showed lower basal AKT activity in WAT depots compared to WT mice, which appeared to be mitigated in KO mice fed a flax diet. Collectively, our work suggests that a *Fads2* deficiency limits lipid storage by altering TAG/fatty acid cycling and reducing basal insulin signaling in WAT depots. Moreover, these changes appear to be independent of the *Fads2* deficiency–induced disruption in EPA and DHA synthesis.

In accordance with previous studies (9, 22, 23), *Fads2* KO mice showed lower body weight, smaller WAT depots, and reduced adipocyte size compared to WT mice. Moreover, we observed that these reductions were similar in both lard- and flax-fed mice. Evidence from prior animal work has typically shown that EPA and DHA supplementation reduces lipid accumulation in WAT (33, 34, 35, 36, 37). *In vitro* studies using 3T3-L1 cells also showed reduced TAG content with EPA/DHA treatment (24, 25, 38). However, we observed that *Fads2* KO mice fed a flax diet had lower fat mass and adipocyte size compared to WT mice, despite the reductions in EPA and DHA content in WAT depots. In addition, similar levels of reduction were also found in mice fed a lard diet with minimal changes of N-3 PUFA content in WAT. This suggests that D6D is capable of influencing WAT lipid storage independent of changes in N-3 LC-PUFA content.

WAT is a key player in the regulation of whole-body metabolism. Reductions in body weight and fat mass are generally associated with increased resting energy expenditure and improved insulin sensitivity (39, 40, 41, 42). A previous study by Stoffel *et al.* reported reduced insulin sensitivity and resting energy expenditure in *Fads2* KO mice compared to WT mice when fed a regular diet free of N-3 LC-PUFA but containing ALA and LA to avoid essential fatty acid deficiency. However, the impairment in glucose tolerance in the *Fads2* KO mice was surprising given that these animals weighed less and had smaller adipocytes (23), *i.e*., characteristics that are generally associated with improved metabolic health. In contrast, our study did not observe any differences in energy expenditure between WT and KO mice fed lard or flax diets. Furthermore, our ITT and GTT data did not achieve statistical significance, although glucose tolerance trended lower in Lard-KO mice compared to Lard-WT mice (*P*_AUC_ = 0.09). Together, our findings suggest that the lower body weight and fat mass observed in *Fads2* KO mice is not attributed to changes in energy expenditure and that the *Fads2* genotype may have minimal impact on whole-body insulin sensitivity in a low-fat feeding context. Interestingly, our data suggest a possible reduction in feed efficiency in KO mice that may contribute to their lower body. Future experiments measuring fecal energy content may reveal differences in energy extraction between WT and KO mice.

Perturbations in lipid metabolism that affect TAG/fatty acid cycling in WAT are strongly associated with metabolic disease (43). As such, examining whether a *Fads2* deficiency impacts pathways that control TAG content in WAT, such as lipogenesis, lipolysis, and fatty acid re-esterification, is of particular relevance. Despite the reduction in the weight of both WAT depots, we observed an increase in various lipogenic markers. However, these findings align with the lower levels of EPA and DHA in KO mice, since EPA and DHA were reported to suppress lipogenic gene expression in animal studies (26, 35) and cell culture (24, 38). Therefore, the higher levels of lipogenic markers observed in *Fads2* KO mice could be partially attributed to the lower EPA/DHA levels in WAT. When comparing WT and KO mice, animals fed the flax diet showed much larger changes in EPA and DHA content compared to mice fed the lard diet; however, the reduction in lipogenic markers were similar between the two diet groups. This suggests that the influence of the *Fads2* genotype on WAT lipogenesis extends beyond its effects on EPA and/or DHA content.

In addition to lipogenesis, an increase in lipolysis can also cause a reduced lipid content in WAT (14). We observed higher ATGL protein level and HSL activation, as well as higher serum NEFA, in *Fads2* KO mice, which aligns with the lower WAT mass observed in these mice. WAT lipolysis is tightly controlled by insulin signaling via both transcriptional and posttranslational regulation (14). Specifically, insulin inhibits lipolysis via activation of the PI3K–PDK–AKT pathway, promoting cAMP hydrolysis. The reduction in cellular cAMP levels inactivates PKA, which lowers HSL phosphorylation (44). In addition, reduced PKA activity is also associated with reduced ATGL activity (45). Moreover, insulin inhibits *Atgl* expression via reduced activities of transcription factors such as FOXO1 and STAT5 (46, 47). However, *Hsl* transcription is regulated by insulin-independent factors, such as retinoid X receptor, liver X receptor alpha, and SREBPs (48, 49). Since we found that a *Fads2* deficiency led to changes in HSL phosphorylation, but not in *Hsl* transcription, we speculate that the increase in markers of lipolysis stemmed from changes in the WAT insulin signaling pathway. In alignment, we found a reduction in both pAKT^Thr308^/AKT and pAKT^Ser473^/AKT ratios, indicating a reduction in the basal activity of this canonical pathway that may explain the increase in lipolysis. Since serum insulin concentrations were similar between groups, our findings may indicate an early impairment in WAT insulin sensitivity with a *Fads2* deficiency. In addition, we recently reported no changes in markers of insulin signaling in the basal state in skeletal muscle of these same mice (50). Together, our results suggest that a *Fads2* deficiency may initially promote insulin resistance in adipose tissues, even in the context of a low-fat diet, before this is manifested in other tissues or at the whole-body level.

Interestingly, KO mice fed a lard diet showed a consistent reduction in the two measured pAKT/AKT ratios in both WAT depots when compared to WT mice, whereas mice fed a flax diet showed no differences between WT and KO except for the pAKT^Thr308^/AKT ratio in iWAT. This suggests that ALA may potentially compensate for the impairment in WAT insulin signaling caused by a *Fads2* deficiency. Although evidence from animal feeding studies suggests that an ALA-enriched diet improves whole-body and muscle insulin sensitivity (51, 52, 53), these studies were unable to determine whether the improvement was associated with ALA or endogenously produced EPA/DHA. However, using *Fads2* KO mice fed a flax diet, we showed that ALA alone appeared capable of regulating basal insulin signaling in WAT. Interestingly, several human studies found a positive association between adipose tissue ALA content and insulin sensitivity in healthy adult men and women assessed by HOMA-IR (54) and in elderly men measured by a euglycemic clamp (55), whereas EPA and DHA levels showed no or even inverse associations in these studies. The effect of individual N-3 PUFA on insulin sensitivity warrants further investigation.

Critical to the regulation of WAT TAG/fatty acid cycling is the generation of glycerol-3 phosphate via glyceroneogenesis. PEPCK1 is a key enzyme involved in glyceroneogenesis and fatty acid re-esterification in WAT (56). During fasting, reduced serum insulin allows for an increase in adipocyte cAMP levels, thereby promoting TAG lipolysis to release NEFA and glycerol into circulation (57). Fatty acid release is restricted, as a significant amount of NEFA is taken back up by adipocytes, activated by fatty acyl-CoA synthetase, and re-esterified to glycerol (56, 58). While adipocytes have low glycerol kinase activity (59), an increased cAMP can promote PEPCK1, thus leading to the production of local intracellular glycerol from oxaloacetate to support TAG synthesis (57, 59). Elevated lipolysis is reflected by increased NEFA in circulation. Under this increased lipolysis, PEPCK in adipocytes is activated and attenuates NEFA release to circulation by promoting glycerol-3 phosphate production to support fatty acid re-esterification. The *Fads2* KO mice in our study showed a higher serum NEFA/glycerol level but with higher PEPCK1 in WAT, reflecting increased lipolysis in *Fads2* KO mice. Interestingly, Flachs *et al.* (60) reported a connection between TAG/fatty acid cycling and lipogenesis in WAT in cold-exposed mice, suggesting that a reduction in fatty acid re-esterification may be partially offset by an increase in lipogenesis to support TAG synthesis. This provides additional support for our findings showing higher lipogenesis and lipolysis, concomitant with lower fatty acid re-esterification.

We recognize the following limitations in the present study that require further investigations. First, the present study analyzed mRNA and protein expression to assess changes in lipogenesis, lipolysis, and fatty acid re-esterification in WAT. Although we measured numerous markers, we acknowledge that these are not direct measurements of lipid turnover in WAT. Therefore, future studies using adipose tissue organ culture would complement our in vivo study and allow for a more controlled investigation of cellular functions and processes. Moreover, such an ex vivo experimental system would enable us to measure both glycerol and NEFA release directly from WAT, as well as their intratissue levels. Additionally, measuring lipoprotein lipase activity in these depots will provide insight into fatty acid uptake, which comprises another important facet of WAT lipid handling. Second, low-fat diets were used in the present study; therefore, our conclusions cannot be generalized to animals fed a high-fat diet or a Western diet. Third, other pathways may also contribute to the lower fat mass and adipocyte size in *Fads2* KO mice that may be of interest for future investigations, such as lipid oxidation in the mitochondria, β-adrenergic receptor-mediated PKA activation, and cGMP-mediated PKG activation of lipolysis. In addition, emerging evidence suggests that lipophagy (autophagic degradation of lipid) has an essential contribution to lipid metabolism in adipose tissue (61, 62), which will also be worthwhile to investigate in *Fads2* KO mice. Finally, due to the difficulty in generating KO mice, additional animals were not obtained which would have enabled us to measure markers of WAT insulin signaling under insulin-stimulated conditions, as well as investigate these processes in female animals.

In conclusion, the present study revealed a novel role of *Fads2* on TAG/fatty acid cycling in WAT. A *Fads2* deficiency limits lipid storage in WAT via increased lipolysis which was partially associated with a reduced insulin signaling. Also, the *Fads2* deficiency-induced changes in EPA/DHA composition in WAT appear to have little-to-no influence on the observed changes in *Fads2* KO mice, indicating that the *Fads2* gene itself plays a role in regulating TAG turnover in WAT. Collectively, the current study revealed a previously unrecognized role in vivo for *Fads2* and its ability to influence TAG/fatty acid cycling in WAT.

## Data availability

The datasets generated and analyzed for the current study are available from the corresponding author on reasonable request. All data generated and analyzed during this study are included in this published article.

## Supplemental data

This article contains supplemental data.

## Conflict of interest

The authors declare that they have no conflicts of interest with the contents of this article.
