# Supplementary material for: Delta-6 desaturase (Fads2) deficiency alters triacylglycerol/fatty acid cycling in murine white adipose tissue
Source: J Lipid Res. 2023 Apr 19;64(6):100376. doi: 10.1016/j.jlr.2023.100376 (PMC10323924; doi:10.1016/j.jlr.2023.100376)

SUPPLEMENTAL INFORMATION:

**Delta-6 desaturase (*Fads2*) deficiency alters triacylglycerol / fatty acid cycling in murine white adipose tissue**

Chenxuan Wang^a^, Barbora Hucik^a^, Ousseynou Sarr^a^, Liam H. Brown^a^, Kyle R.D. Wells^b^, Keith R. Brunt^b,^ Manabu T. Nakamura^c^, Ewa Harasim-Symbor^d^, Adrian Chabowski^d^, David M. Mutch^a,^*

^a^ Department of Human Health and Nutritional Sciences, University of Guelph, Guelph, ON, Canada

^b^ Department of Pharmacology, Dalhousie University, Saint John, NB, Canada

^c^ Department of Food Science and Human Nutrition, University of Illinois at Urbana-Champaign, Urbana, IL, USA

^d^ Department of Physiology, Medical University of Bialystok, Bialystok, Poland

**Supplemental Table S1. Composition of experimental diets.** Weight % in grams (gm%) does not equal 100 as these values do not account for minerals and non-soluble fibre in the diet. *ARASCO refers to a mixture of a high-arachidonic acid oil extracted from the fungus Mortierella alpine and high-oleic sunflower seed oil.

|  | CAT#: D16090607  Lard | | CAT#: D12041404  Flax | |
| --- | --- | --- | --- | --- |
|  | **gm%** | **kcal%** | **gm%** | **kcal%** |
| Protein | 20 | 20 | 20 | 20 |
| Carbohydrate | 64 | 64 | 64 | 64 |
| Fat | 7 | 16 | 7 | 16 |
| *Total* |  | *100* |  | *100* |
| *kcal/gm* | *4* |  | *4* |  |
| Ingredient | **gm** | **kcal** | **gm** | **kcal** |
| Casein | 200 | 800 | 200 | 800 |
| L-Cystine | 3 | 12 | 3 | 12 |
| Corn Starch | 397.486 | 1590 | 397.486 | 1590 |
| Maltodextrin 10 | 132 | 528 | 132 | 528 |
| Sucrose | 100 | 400 | 100 | 400 |
| Cellulose, BW200 | 50 | 0 | 50 | 0 |
| Lard | 66.25 | 596 | 0 | 0 |
| Flaxseed Oil | 0 | 0 | 66.25 | 596 |
| ARASCO* (40% ARA) | 3.75 | 34 | 3.75 | 34 |
| t-Butylhydroquinone | 0.014 | 0 | 0.014 | 0 |
| Mineral Mix S10022G | 35 | 0 | 35 | 0 |
| Vitamin Mix V10037 | 10 | 40 | 10 | 40 |
| Choline Bitartrate | 2.5 | 0 | 2.5 | 0 |
| *Total* | *1000.05* | *4000* | *1000.05* | *4000* |
| Fatty Acid | **gm/4000 kcal** | | **gm/4000 kcal** | |
| C12, Lauric | 0.1 | | 0 | |
| C14, Myristic | 0.8 | | 0 | |
| C15 | 0.1 | | 0 | |
| C16, Palmitic | 13.3 | | 3.2 | |
| C16:1, Palmitoleic | 0.9 | | 0 | |
| C17 | 0.2 | | 0 | |
| C18, Stearic | 7.3 | | 2.4 | |
| C18:1, Oleic | 22.2 | | 12.4 | |
| C18:2, Linoleic | 16.4 | | 10.9 | |
| C18:3, Linolenic | 1 | | 36.6 | |
| C20, Arachidic | 0.1 | | 0 | |
| C20:1, | 0.4 | | 0 | |
| C20:2 | 0.5 | | 0 | |
| C20:3, n6 | 0.1 | | 0 | |
| C20:4, Arachidonic | 1.7 | | 1.5 | |
| C20:5, Eicosapentaenoic | 0 | | 0 | |
| C22:1, Erucic | 0.1 | | 0.1 | |
| C22:5, Docosapentaenoic | 0.1 | | 0 | |
| C22:6, Docosahexaenoic | 0 | | 0 | |
| *Total* | *65.4* | | *67* | |

Supplemental Table S2. Primer sequences for RT-qPCR.

|  | Forward | Reverse |
| --- | --- | --- |
| *Ppar-γ* | TGCTGTTATGGGTGAAACTCTG | CTGTGTCAACCATGGTAATTTCTT |
| *Fabp4* | TCGACCACAATAAAGAGAAAACG | CTTGTGGAAGTCACGCCTTT |
| *C/ebpα* | TCTCCCCTAGTTGTCCAAGG | GGGTGGGGAAGCCTAAGTC |
| *Srepb1* | ATCGGCGCGGAAGCTGTCGGGGTAGCGTC | ACTGTCTTGGTTGTTGATGAGCTGGAGCAT |
| *Scd1* | GAGACCTGATACCTAACACTCTGTCA | GATGTGATGTTTTCTTCTAGACTTTCC |
| *Fasn* | CCAAATCCAACATGGGACA | TGCTCCAGGGATAACAGCA |
| *Acc* | GCGTCGGGTAGATCCAGTT | CTCAGTGGGGCTTAGCTCTG |
| *Dgat2* | GGCGCTACTTCCGAGACTAC | TGGTCAGCAGGTTGTGTGTC |
| *Atgl* | TGACCATCTGCCTTCCAGA | TGTAGGTGGCGCAAGACA |
| *Hsl* | CACAAAGGCTGCTTCTACGG | GGAGAGAGTCTGCAGGAACG |
| *Pepck1* | GGAGTACCCATTGAGGGTATCAT | GCTGAGGGCTTCATAGACAAG |
| *Nono* | CCCCACCAATACCTGCAA | TTCAGGTCAATAGTCAAGCCTTC |

Supplemental Table S3. Fatty acid content (μmol/g tissue) in eWAT from wildtype (WT) and *Fads2* knockout (KO) mice fed a lard or flax diet.

|  | | **Lard-WT** | **Lard-KO** | **Flax-WT** | **Flax-KO** |
| --- | --- | --- | --- | --- | --- |
| **14:0** | DAG | 0.3±0.2 | 0.3±0.1 | 0.4±0.2 | 0.4±0.2 |
|  | TAG | 26.4±3.5 | 33.8±8.5 | 23.3±3.0 | 34.8±9.7 |
|  | PL | 0.3±0.1 | 0.2±0.1 | 0.2±0.1 | 0.3±0.0 |
|  | Total | 27.0±3.6 | 34.3±8.6 | 23.9±3.1 | 35.5±9.8 |
| **16:0** | DAG | 1.7±0.6 | 1.6±0.4 | 2.0±0.7 | 1.9±0.5 |
|  | TAG | 395.4±44.0 | 422.3±59.4 | 359.6±64.2 | 416.9±80.7 |
|  | PL | 1.3±0.7 | 1.1±0.8 | 1.5±0.6 | 2.0±0.4 |
|  | Total | 398.4±44.4 | 424.9±60.3 | 362.9±64.3 | 420.7±80.4 |
| **16:1** | DAG | 0.6±0.1 | 0.6±0.1 | 0.6±0.2 | 0.7±0.2 |
|  | TAG | 174.7±31.9 | 230.1±65.2 | 140.7±20.9 | 259.4±48.8 |
|  | PL | 0.4±0.3 | 0.3±0.1 | 0.3±0.2 | 0.4±0.1 |
|  | Total | 175.6±31.9 | 230.9±65.3 | 141.6±21.0 | 260.4±48.9 |
| **18:0** | DAG | 0.4±0.2 | 0.4±0.1 | 0.5±0.3 | 0.5±0.3 |
|  | TAG | 36.1±4.9 | 35.8±6.7 | 29.7±6.2 | 27.5±9.3 |
|  | PL | 1.0±0.1 | 0.8±0.2 | 1.0±0.2 | 1.2±0.4 |
|  | Total | 37.5±5.1 | 36.9±6.7 | 31.3±6.2 | 29.2±9.1 |
| **18:1n9** | DAG | 1.9±0.5 | 1.8±0.4 | 2.0±0.9 | 1.6±0.3 |
|  | TAG | 732.7±101.4 | 834.9±114.3 | 594.3±76.0 | 712.7±78.3 |
|  | PL | 0.5±0.2 | 0.5±0.3 | 0.7±0.3 | 1.0±0.3 |
|  | Total | 735.1±101.7 | 837.2±114.4 | 596.9±76.1 | 715.3±78.6 |
| **18:2n6** | DAG | 0.7±0.2 | 0.6±0.2 | 0.9±0.4 | 0.7±0.2 |
|  | TAG | 209.7±28.5 | 229.0±34.8 | 216.7±28.4 | 228.1±35.6 |
|  | PL | 0.5±0.1 | 0.7±0.2 | 0.7±0.1 | 0.7±0.1 |
|  | Total | 210.9±28.7 | 230.4±34.9 | 218.3±28.7 | 229.5±35.9 |
| **18:3n3** | DAG | 0.1±0.0 | 0.1±0.0 | 1.1±0.5 | 1.0±0.3 |
|  | TAG | 18.2±8.3 | 24.4±7.8 | 460.8±61.1 | 553.3±65.3 |
|  | PL | 0.1±0.0 | 0.1±0.0 | 0.2±0.1 | 0.2±0.1 |
|  | Total | 18.4±8.4 | 24.5±7.9 | 462.1±61.5 | 554.5±65.6 |
| **20:0** | DAG | ND | ND | ND | ND |
|  | TAG | 1.0±0.4 | 1.2±0.3 | 1.2±0.4 | 1.5±0.6 |
|  | PL | 0.1±0.1 | 0.1±0.0 | 0.1±0.1 | 0.1±0.0 |
|  | Total | 1.2±0.4 | 1.3±0.3 | 1.3±0.4 | 1.7±0.6 |
| **20:4n6** | DAG | 0.1±0.0 | 0.1±0.0 | 0.1±0.0 | 0.1±0.0 |
|  | TAG | 8.6±0.7 | 8.1±1.6 | 6.6±1.2 | 8.9±1.4 |
|  | PL | 0.6±0.1 | 0.6±0.1 | 0.6±0.2 | 0.7±0.2 |
|  | Total | 9.3±0.7 | 8.8±1.7 | 7.2±1.3 | 9.8±1.5 |
| **20:5n3** | DAG | ND | ND | ND | ND |
|  | TAG | 0.3±0.1 | 0.3±0.1 | 2.6±0.6 | 0.3±0.1 |
|  | PL | 0.3±0.1 | 0.2±0.0 | 0.2±0.1 | 0.2±0.1 |
|  | Total | 0.6±0.1 | 0.4±0.1 | 2.8±0.6 | 0.5±0.2 |
| **22:0** | DAG | ND | ND | ND | ND |
|  | TAG | 0.4±0.1 | 1.0±0.4 | 0.3±0.2 | 0.9±0.4 |
|  | PL | ND | ND | ND | ND |
|  | Total | 0.4±0.1 | 1.0±0.4 | 0.3±0.2 | 1.0±0.4 |
| **22:6n3** | DAG | ND | ND | ND | ND |
|  | TAG | 1.4±0.2 | 0.3±0.1 | 5.3±1.3 | 0.2±0.1 |
|  | PL | 0.4±0.1 | 0.3±0.1 | 0.4±0.1 | 0.4±0.2 |
|  | Total | 1.7±0.2 | 0.6±0.2 | 5.8±1.3 | 0.6±0.2 |
| **24:0** | DAG | ND | ND | ND | ND |
|  | TAG | 0.2±0.1 | 0.1±0.1 | 0.2±0.2 | 0.2±0.2 |
|  | PL | ND | ND | ND | ND |
|  | Total | 0.2±0.1 | 0.2±0.1 | 0.2±0.2 | 0.2±0.2 |
| **24:1** | DAG | ND | ND | ND | ND |
|  | TAG | 0.1±0.1 | 0.1±0.1 | 0.2±0.2 | 0.5±0.1 |
|  | PL | ND | ND | ND | ND |
|  | Total | 0.1±0.1 | 0.1±0.1 | 0.2±0.2 | 0.5±0.1 |
| **Total DAG** | | 5.9±1.9 | 5.7±1.5 | 7.6±3.3 | 7.1±2.1 |
| **Total TAG** | | 1605.2±224.2 | 1821.2±299.5 | 1841.6±264.0 | 2245.2±330.6 |
| **Total PL** | | 5.5±2.2 | 4.9±2.0 | 5.9±2.1 | 7.1±1.9 |

ND: not detected or below 0.01μmol/g; DAG: diacylglycerol; TAG: triacylglycerol; PL: phospholipid. All data is reported as mean ± SD (n=6–8 mice/group).

Supplemental Table S4. Fatty acid content (μmol/g tissue) in iWAT from wildtype (WT) and *Fads2* knockout (KO) mice fed a lard or flax diet.

|  | | **Lard-WT** | **Lard-KO** | **Flax-WT** | **Flax-KO** |
| --- | --- | --- | --- | --- | --- |
| **14:0** | DAG | 0.2±0.0 | 0.3±0.1 | 0.3±0.0 | 0.3±0.0 |
|  | TAG | 30.6±5.7 | 29.2±9.8 | 23.7±3.3 | 30.4±8.5 |
|  | PL | 0.3±0.1 | 0.2±0.0 | 0.2±0.0 | 0.2±0.1 |
|  | Total | 31.1±5.7 | 29.7±9.9 | 24.2±3.3 | 30.9±8.4 |
| **16:0** | DAG | 1.3±0.2 | 1.4±0.5 | 1.3±0.2 | 1.4±0.3 |
|  | TAG | 409.9±36.3 | 335.7±10.5 | 304.0±43.1 | 345.6±88.4 |
|  | PL | 1.2±0.3 | 1.3±0.5 | 1.3±0.4 | 1.0±0.3 |
|  | Total | 412.5±36.5 | 338.4±105.5 | 306.6±42.9 | 348.1±886.1 |
| **16:1** | DAG | 0.4±0.0 | 0.5±0.2 | 0.4±0.1 | 0.5±0.1 |
|  | TAG | 212.5±37.8 | 186.9±65.9 | 146.8±41.5 | 180.6±51.4 |
|  | PL | 0.3±0.1 | 0.3±0.1 | 0.3±0.0 | 0.3±0.1 |
|  | Total | 213.2±37.9 | 187.8±66.1 | 147.44±41.6 | 181.3±51.4 |
| **18:0** | DAG | 0.3±0.1 | 0.4±0.1 | 0.3±0.1 | 0.3±0.1 |
|  | TAG | 30.9±10.3 | 28.7±10.1 | 24.4±6.1 | 28.5±12.6 |
|  | PL | 1.2±0.3 | 1.1±0.4 | 1.2±0.5 | 1.1±0.6 |
|  | Total | 32.4±10.2 | 30.1±10.6 | 25.9±6.5 | 29.9±12.8 |
| **18:1n9** | DAG | 1.5±0.2 | 1.7±0.6 | 1.2±0.4 | 1.5±0.2 |
|  | TAG | 887.9±99.3 | 805.7±218.7 | 646.7±126.1 | 679.1±157.9 |
|  | PL | 1.0±0.5 | 1.1±0.5 | 0.7±0.3 | 0.7±0.3 |
|  | Total | 890.3±99.8 | 808.5±219.5 | 648.6±125.9 | 681.3±157.9 |
| **18:2n6** | DAG | 0.6±0.1 | 0.7±0.2 | 0.6±0.2 | 0.6±0.1 |
|  | TAG | 243.9±32.8 | 200.5±51.4 | 217.1±44.4 | 199.9±56.1 |
|  | PL | 0.8±0.2 | 0.9±0.4 | 0.7±0.3 | 0.8±0.4 |
|  | Total | 245.3±32.9 | 202.1±51.8 | 218.4±44.2 | 201.4±55.9 |
| **18:3n3** | DAG | 0.05±0.00 | 0.06±0.00 | 0.8±0.2 | 0.8±0.2 |
|  | TAG | 15.5±2.3 | 25.5±10.0 | 441.7±97.4 | 401.9±115.3 |
|  | PL | 0.2±0.1 | 0.2±0.0 | 0.2±0.1 | 0.2±0.1 |
|  | Total | 15.8±2.3 | 25.7±10.1 | 442.6±97.5 | 402.9±115.4 |
| **20:0** | DAG | ND | ND | ND | ND |
|  | TAG | 0.9±0.1 | 1.1±0.4 | 1.0±0.2 | 1.3±0.3 |
|  | PL | 0.1±0.0 | 0.1±0.0 | 0.1±0.0 | 0.1±0.0 |
|  | Total | 1.0±0.1 | 1.2±0.4 | 1.1±0.2 | 1.4±0.3 |
| **20:4n6** | DAG | ND | ND | ND | ND |
|  | TAG | 8.2±1.7 | 5.7±2.3 | 6.1±1.1 | 6.3±1.9 |
|  | PL | 0.9±0.3 | 0.9±0.4 | 0.7±0.4 | 0.9±0.5 |
|  | Total | 9.2±1.8 | 6.7±2.5 | 6.8±0.8 | 7.2±1.8 |
| **20:5n3** | DAG | ND | ND | ND | ND |
|  | TAG | 0.7±0.3 | 0.4±0.2 | 3.4±0.9 | 0.5±0.2 |
|  | PL | 0.2±0.0 | 0.1±0.1 | 0.1±0.0 | 0.1±0.0 |
|  | Total | 0.9±0.3 | 0.5±0.2 | 3.5±0.9 | 0.7±0.2 |
| **22:0** | DAG | ND | ND | ND | ND |
|  | TAG | 0.3±0.1 | ND | ND | 0.5±0.2 |
|  | PL | ND | ND | ND | ND |
|  | Total | 0.3±0.1 | ND | ND | 0.5±0.2 |
| **22:6n3** | DAG | ND | ND | ND | ND |
|  | TAG | 1.3±0.5 | ND | 4.9±1.1 | 0.5±04 |
|  | PL | 0.3±0.1 | 0.2±0.0 | 0.3±0.1 | 0.2±0.0 |
|  | Total | 1.6±0.5 | 0.2±0.0 | 5.3±1.1 | 0.7±0.4 |
| **24:0** | DAG | ND | ND | ND | ND |
|  | TAG | ND | ND | ND | ND |
|  | PL | ND | ND | ND | ND |
|  | Total | ND | ND | ND | ND |
| **24:1** | DAG | ND | ND | ND | ND |
|  | TAG | 0.2±0.0 | ND | ND | 0.4±0.2 |
|  | PL | ND | ND | ND | ND |
|  | Total | 0.2±0.0 | ND | ND | 0.4±0.2 |
| **Total DAG** | | 4.6±0.5 | 5.2±1.6 | 5.1±1.0 | 5.7±1.0 |
| **Total TAG** | | 1842.7±192.0 | 1746.5±337.7 | 1642.1±502.0 | 1875.6±439.2 |
| **Total PL** | | 6.3±1.6 | 6.6±2.4 | 5.4±2.0 | 5.5±2.3 |

ND: not detected or below 0.01μmol/g; DAG: diacylglycerol; TAG: triacylglycerol; PL: phospholipid. All data is reported as mean ± SD (n=6–8 mice/group).

**Supplemental Figure S1. Tissue weights and adipocyte sizes of inguinal white adipose tissue (iWAT) in wildtype (WT) and *Fads2* knockout (KO) mice fed a Lard or Flax diet.** (A) iWAT tissue weight was calculated as % of body weight. (B) iWAT adipocyte size and representative images. All data is presented as mean ± SD (n=8–12 mice/group). Data was analyzed by 2-way ANOVA for main effects (P_diet_ & P_genotype_) and interaction (P_Interaction_) followed by a Tukey's post-hoc test. Different letters indicate a significant difference (P < 0.05) between groups.


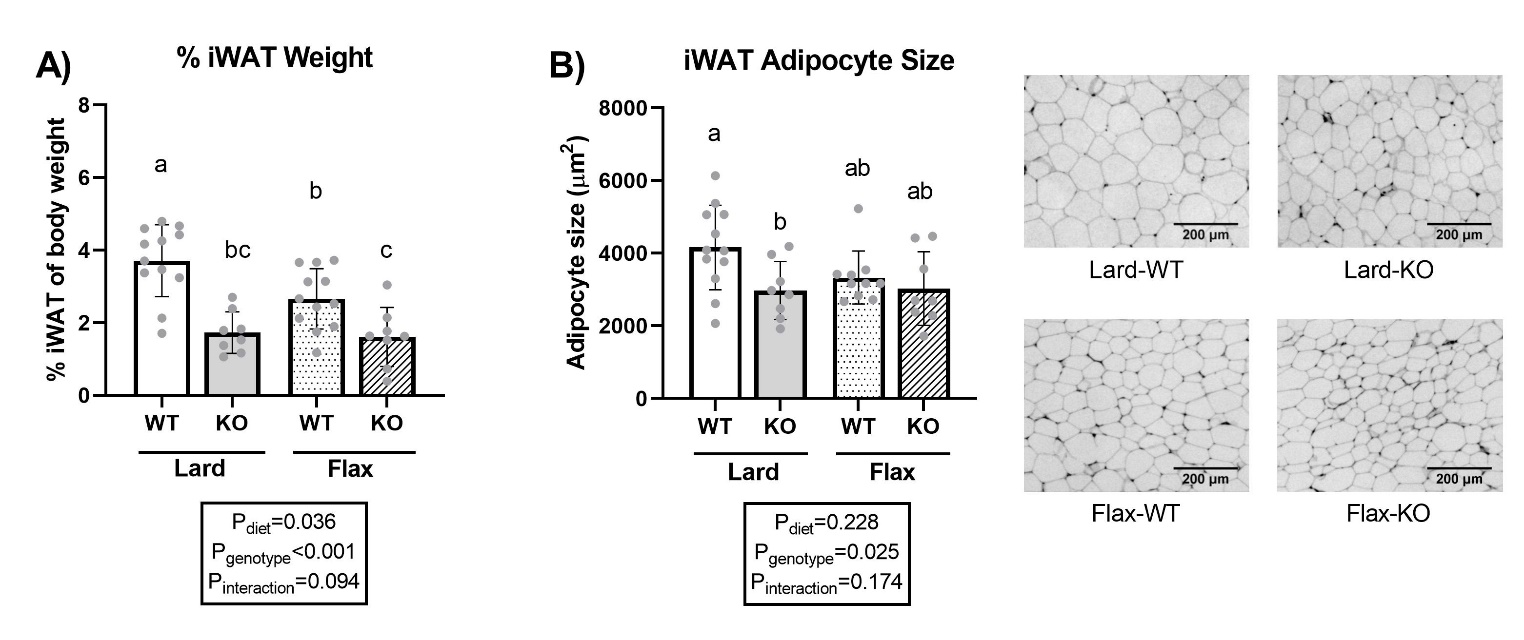


Supplemental Figure S2. N-3 and N-6 fatty acid composition in inguinal white adipose tissue (iWAT) from wildtype (WT) and *Fads2* knockout (KO) mice fed a Lard or Flax diet. The complete fatty acid composition of phospholipid (PL), triacylglycerol (TAG), and diacylglycerol (DAG) fractions is reported in Supplemental Table S4. Total µmol per g tissue of (A) α-linolenic acid (ALA), (B) eicosapentaenoic acid (EPA), (C) docosahexaenoic acid (DHA), (D) linoleic acid (LA), and (E) arachidonic acid (AA) were calculated by summing the values in PL, TAG and DAG fractions for each fatty acid. N-3 and N-6 polyunsaturated fatty acids (PUFA) in PL (F) and TAG (G) are presented as % of total fatty acids in each respective fraction. All data is reported as mean ± SD (n=6–8 mice/group). Data was analyzed by one-way ANOVA with Tukey’s post-hoc test. Different letters indicate a significant difference (P < 0.05) between groups.


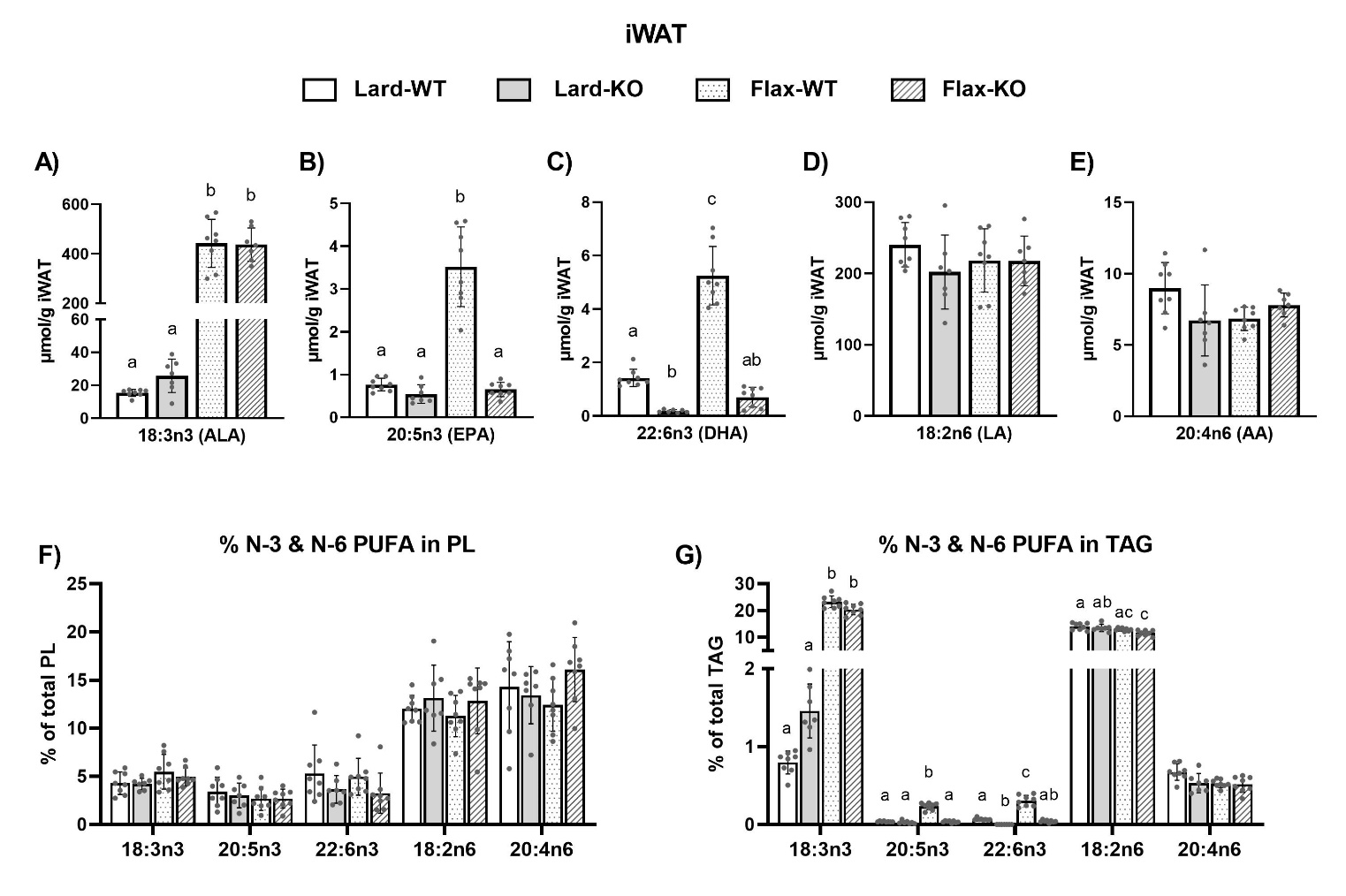


Supplemental Figure S3. Lipogenic markers in inguinal white adipose tissue (iWAT) from wildtype (WT) and *Fads2* knockout (KO) mice fed a Lard or Flax diet. A) RT-qPCR analysis of markers involved in adipogenesis and lipogenesis. (B) Western blot analyses of markers involved in lipogenesis. Data is presented as fold changes compared to the Lard-WT group. (C) Representative images from Western blotting shown for 3 mice/group. αTubulin is shown as a representative loading control. All data is presented as mean ± SD (n=7–12 mice/group). Data was analyzed by 2-way ANOVA for main effects (P_diet_ & P_genotype_) and interaction (P_Interaction_) followed by a Tukey's post-hoc test. Different letters indicate a significant difference (P < 0.05) between groups.


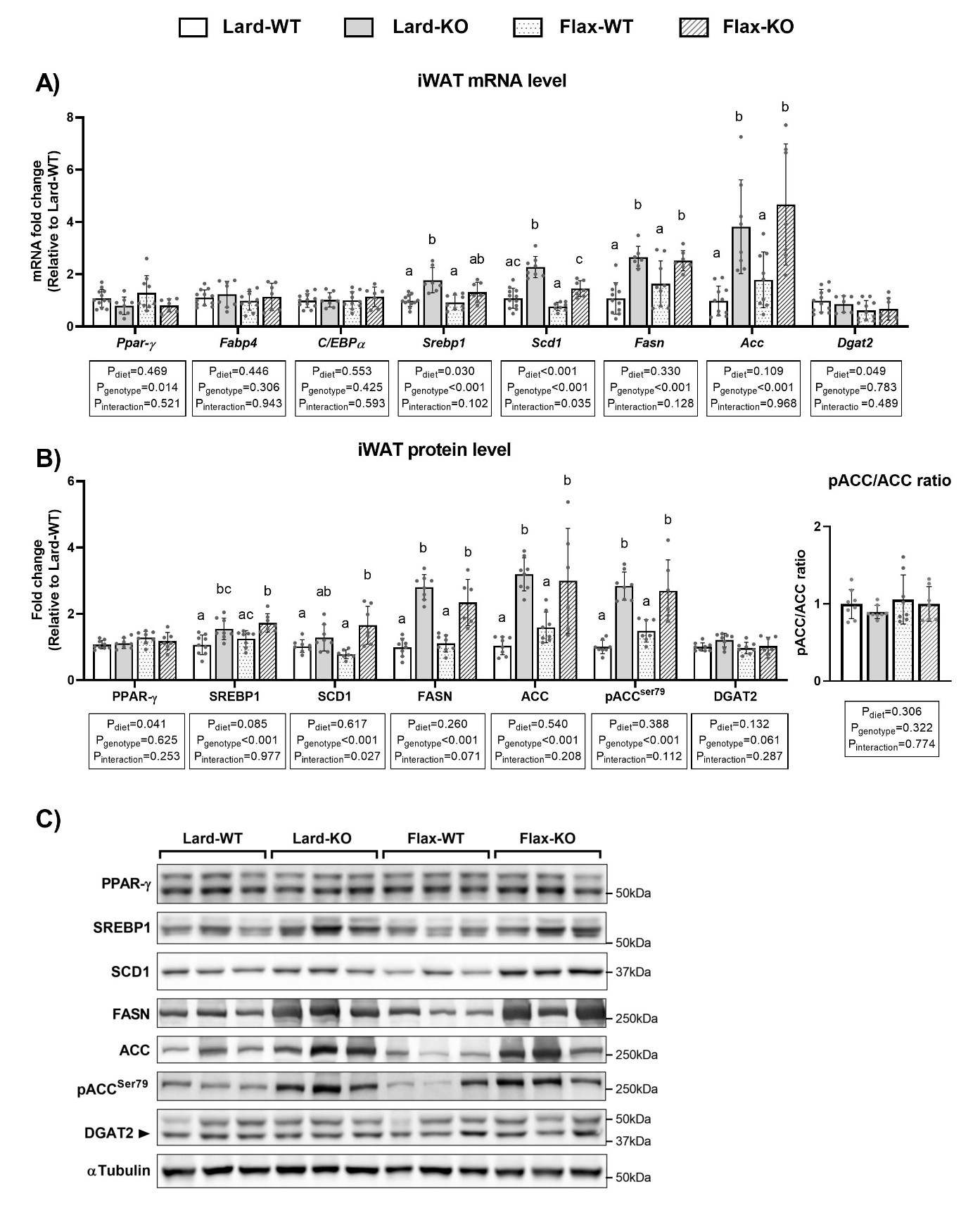


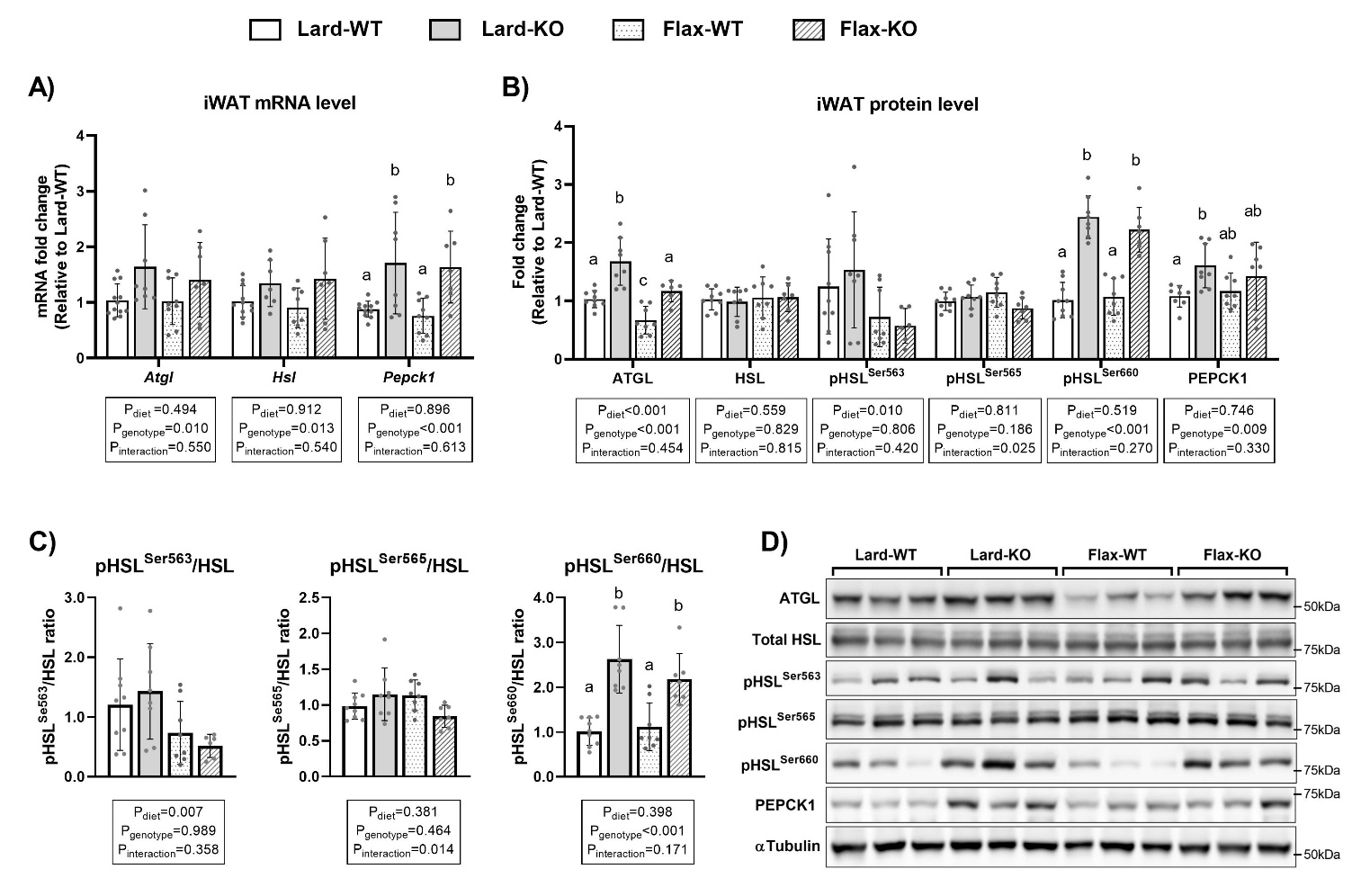
Supplemental Figure S4. Lipolytic markers in inguinal white adipose tissue (iWAT) from wildtype (WT) and *Fads2* knockout (KO) mice fed a Lard or Flax diet. A) RT-qPCR analysis of markers involved in lipolysis. (B) Western blot analyses of markers involved in lipolysis. Data is presented as fold changes against Lard-WT group. (C) Ratios of pHSL to total HSL. (D) Representative images from Western blotting shown for 3 mice/group. αTubulin is shown as a representative loading control and is the same as the αTubulin presented in Figure S3. All data is presented as mean ± SD (n=7–12 mice/group). Data was analyzed by 2-way ANOVA for main effects (P_diet_ & P_genotype_) and interaction (P_Interaction_) followed by a Tukey's post-hoc test. Different letters above bars indicate a significant difference (P < 0.05) between groups.

Supplemental Figure S5. Insulin signaling markers in inguinal white adipose tissue (iWAT) and representative images from both depots. (A) Western blot analyses of two pAKT/AKT ratios in iWAT. Representative images from Western blotting shown for 3 mice/group in (B) iWAT and (C) eWAT. αTubulin is shown as a representative loading control. All data is presented as mean ± SD (n=7–9 mice/group). Data was analyzed by 2-way ANOVA for main effects (P_diet_ & P_genotype_) and interaction (P_Interaction_) followed by a Tukey's post-hoc test. Different letters above bars indicate a significant difference (P < 0.05) between groups.


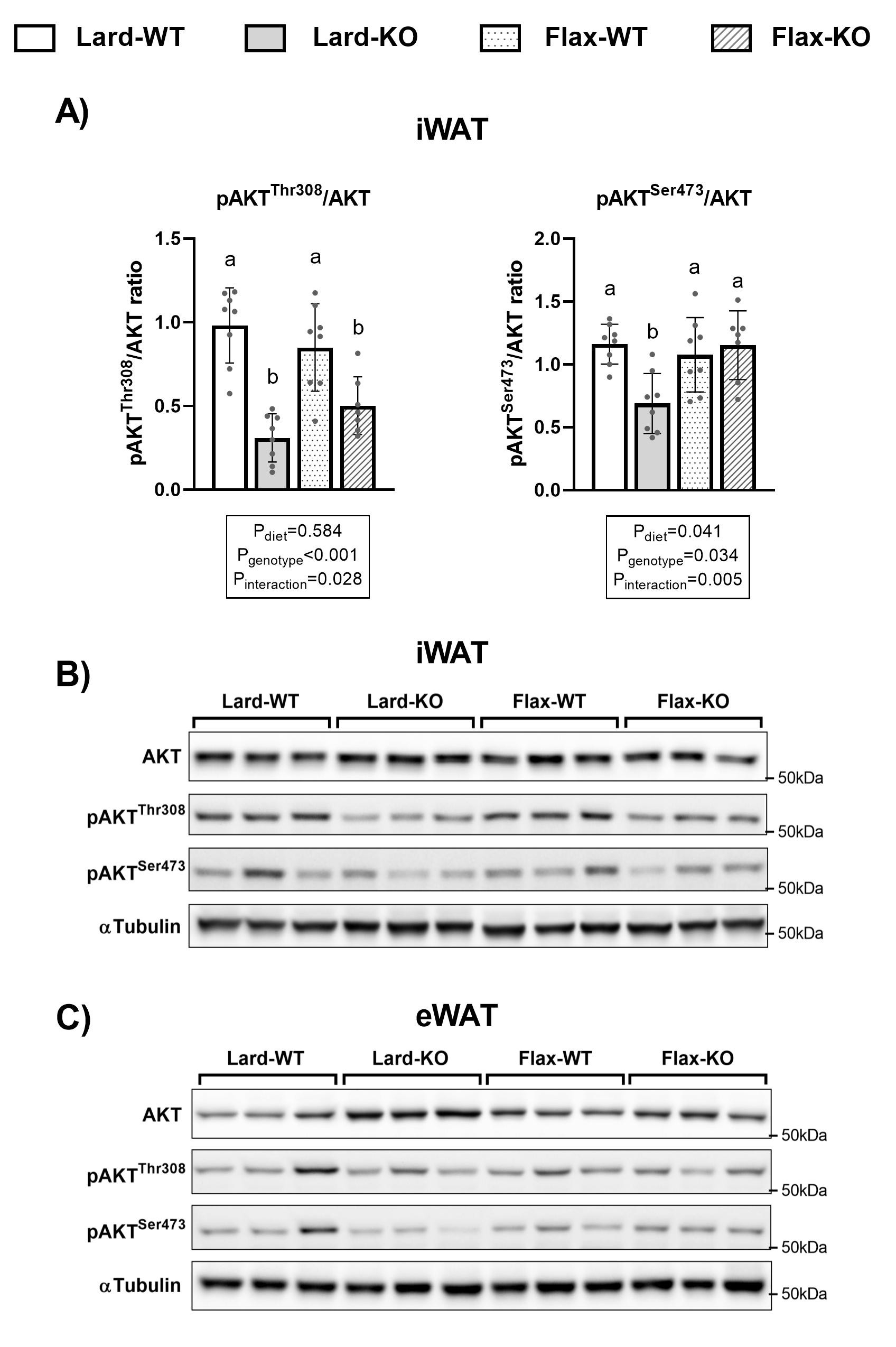

Supplement: Supplemental Tables S1–S4 and Figures S1–S5 [file mmc1.docx]
